# Assessment of Low Global Warming Potential Refrigerants for Drop-In Replacement by Connecting their Molecular Features to Their Performance

**DOI:** 10.1021/acssuschemeng.1c05985

**Published:** 2021-12-07

**Authors:** Carlos G. Albà, Ismail I. I. Alkhatib, Fèlix Llovell, Lourdes F. Vega

**Affiliations:** † Research and Innovation Center on CO_2_ and Hydrogen (RICH Center), Chemical Engineering Department, Khalifa University, P.O. Box 127788, Abu Dhabi, United Arab Emirates; ‡ Department of Chemical Engineering, ETSEQ, Universitat Rovira i Virgili (URV), Av. Països Catalans 26, 43007 Tarragona, Spain

**Keywords:** Polar soft-SAFT, Environmentally friendly refrigerants, Drop-in replacements, Vapor compression refrigeration, Molecular structure−thermophysical properties, Hydrofluoroolefins

## Abstract

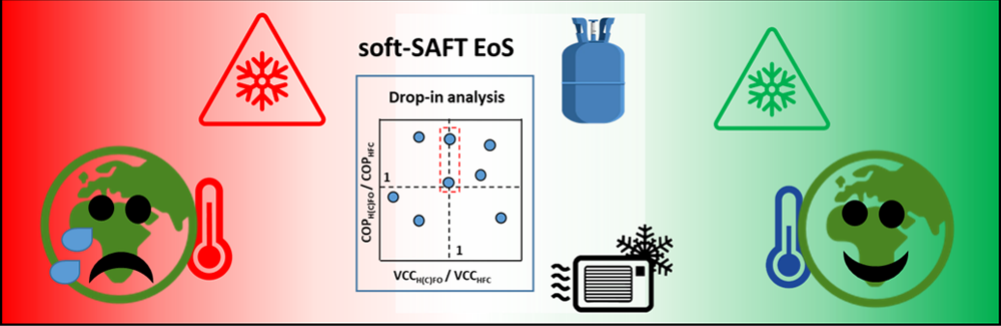

The use of hydrofluorocarbons (HFCs) as an alternative for refrigeration units has grown over the past decades as a replacement to chlorofluorocarbons (CFCs), banned by the Montreal’s Protocol because of their effect on the depletion of the ozone layer. However, HFCs are known to be greenhouse gases with considerable global warming potential (GWP), thousands of times higher than carbon dioxide. The Kigali Amendment to the Montreal Protocol has promoted an active area of research toward the development of low GWP refrigerants to replace the ones in current use, and it is expected to significantly contribute to the Paris Agreement by avoiding nearly half a degree Celsius of temperature increase by the end of this century. We present here a molecular-based evaluation tool aiming at finding optimal refrigerants with the requirements imposed by current environmental legislations in order to mitigate their impact on climate change. The proposed approach relies on the robust polar soft-SAFT equation of state to predict thermodynamic properties required for their technical evaluation at conditions relevant for cooling applications. Additionally, the thermodynamic model integrated with technical criteria enable the search for compatibility of currently used third generation compounds with more eco-friendly refrigerants as drop-in replacements. The criteria include volumetric cooling capacity, coefficient of performance, and other physicochemical properties with direct impact on the technical performance of the cooling cycle. As such, R1123, R1224yd(Z), R1234ze(E), and R1225ye(Z) demonstrate high aptitude toward replacing R134a, R32, R152a, and R245fa with minimal retrofitting to the existing system. The current modeling platform for the rapid screening of emerging refrigerants offers a guide for future efforts on the design of alternative working fluids.

## Abstract 2

A reliable molecular modeling platform is discussed for the predictive assessment of effective drop-in replacement refrigerants incorporating environmental, technical, and thermodynamic insights.

## Introduction

Within the context of sustainable development, climate action is one of the vital topics in the United Nations’ (UN) goals for sustainable future, focusing on increased awareness and mobilization to resist the adverse ecological effects of climate change.^1^ The main culprit responsible for the emergence of this notorious problem was the utilization of highly active ozone depleting chlorofluorocarbons (CFCs) in the refrigeration and cooling industries, leading to their global ban with the enacting of the Montreal Protocol.^2^ At that stage, the dire industrial need for replacements identified third generation refrigerants hydrofluorocarbons (HFCs) as viable options based on their zero ozone depletion potential (ODP) compared to their banned predecessors.^3^ However, an unforeseeable consequence, further aggravating the climate change problem, was their high global warming potential (GWP), as HFCs are potent greenhouse gases, with projected CO_2_-equivalent emissions of 6%–9% of total CO_2_ emissions by 2050.^4^ Once again, stricter environmental legislations^5−9^ were passed to gradually phase out and restrict the usage of third generation refrigerants in upcoming years, targeting replacements with low GWP.

Even though environmentally friendly refrigeration and cooling technologies are being developed such as absorption refrigeration,^10−12^ adsorption refrigeration,^13^ and other novel systems,^14^ the cooling industry is still heavily reliant on the more efficient vapor compression refrigeration cycles (VCRCs) with fluorinated compounds as working fluids. As such, in lieu of the current targets of environmental legislations, the search for alternative refrigerants replacing HFCs for today’s market is solely dictated by eco-friendly properties such as zero ODP, low GWP, and moderate safety-related properties (i.e., flammability, and toxicity), examining a wide array of substances both natural and synthetic as potential replacements to the currently used third generation refrigerants. The resurged interest in the use of well-known natural fluids identified NH_3_, CO_2_, *n*-alkanes, and water as possible working fluids for air-conditioning applications. However, the commercialization of these natural fluids is hindered either by their poor safety-related properties or inability to operate at specific conditions applicable to currently available refrigeration systems. As such, these options are deemed infeasible single-component drop-in replacements, entailing additional retrofitting of refrigeration cycles using third generation refrigerants.^15^

Conversely, synthetic fourth generation refrigerants such as hydrofluoroolefins (HFOs), hydrochlorofluoroolefins (HCFOs), hydrofluoroethers (HFEs), and even selected blends with third generation HFCs, have demonstrated potentiality in replacing currently used third generation working fluids.^16,17^ DuPont and Honeywell developed 2,3,3,3-tetrafluoroprop-1-ene (R1234yf) as an eco-friendly replacement for 1,1,1,2-tetrafluoroethane (R134a) and examined its application for mobile air conditions systems (MAC), identifying its potentiality as a replacement working fluid satisfying environmental and safety constraints, at the price of a reduced system efficiency^18^ and higher cost. In spite of these limitations, R1234yf is already used as a refrigerant for automobile applications in Europe, with a long list of model vehicles using it, following the implementation of the EU Directive 2006/40/EC^6^ in 2013, banning the use of R134a for this application. In a similar fashion, trans-1,3,3,3-tetrafluoroprop-1-ene (R1234ze(E)) was also identified as a viable replacement for R134a in commercial refrigeration cycles and medium temperature heat pumps based on its eco-friendly properties,^19^ yet with the expense of additional capital costs for retrofitting systems to maintain a similar level of performance.^20^ As opposed to natural refrigerants, a drawback associated with fourth generation refrigerants such as R1234yf and R1234ze(E), is their rapid degradation and formation of stable trifluoroacetate (TFA), potentially contaminating surrounding soil and terminal water bodies.^21,22^ However, recent studies established that the expected concentrations of these degradation products will have a negligible impact on the ecosystem.^22,23^ Thus, far, finding a single-component refrigerant encompassing all desirable properties remains an elusive and difficult feat.

Although the previous examples demonstrate the potentiality of some fourth generation synthetic refrigerants with regard to environmental and safety-related properties in line with environmental regulations, the lingering question to be answered is whether these alternatives can satisfy the technical (and safety) demand of the market for different cooling applications. Ascertaining the potentiality of these alternative refrigerants on technical facets is relevant but given a secondary priority as opposed to the more urgent environmental facets, hence their limited availability in the literature.^20,24−30^ Replacing the current commercially used third generation refrigerants with a low GWP alternative is not straightforward and might lead to retrofitting the existing system, resulting in a trade-off, often overlooked, between meeting environmental constraints and incurring additional costs or compromising system performance.^31−34^ To blame for this is the lack of experimental data on the physicochemical properties of alternative refrigerants, essential for their accurate technical evaluation and projection on industrial scale.^35^ This is expected as experimentally obtaining all relevant properties is quite taxing in temporal and monetary terms, given the number of different properties, varying operating conditions, and possible working fluids.

As such, pragmatic and robust computational tools provide a possible remedy to overcome this hurdle, with the capability of rapidly determining potential HFCs replacements, satisfying environmental and technical requirements. With the rise of thermodynamic modeling approaches and computational power, such paradigms have been successfully developed for varying applications such as screening of materials for CO_2_ capture^36−40^ and solvents for other separation processes.^41−44^ The success of these paradigms is entirely dependent on the accuracy and robustness of the chosen thermodynamic model.^24^ In this regard, molecular-based equations of state (EoSs) such as those routed on the statistical associating fluid theory (SAFT)^45^ have become indispensable tools in modeling the thermodynamic behavior of complex fluids. These models have rigorous physical basis from statistical mechanics, enabling them to accurately capture the effect of intermolecular interactions (i.e., dispersive, associating, polar) on macrolevel phenomena. This is an added edge over conventional EoSs, that are incapable of explicitly modeling polar interactions governing the behavior of third and fourth generation refrigerants and other green substances of polar nature.^46−48^ The adoption of SAFT-based models as a central pillar for screening tools has been steadily growing,^49−53^ holding the promise of similar success when applied to screening alternative eco-friendly refrigerants as drop-in replacements under the same operating conditions and technical criteria. This is fueled by the demonstrated success of our group in accurately modeling the holistic thermodynamic behavior of third and fourth generation refrigerants.^54−58^ On another front, the physical basis of SAFT-based models can enable the extraction of microlevel tendencies linked with observable physicochemical properties and technical performance. This fundamental level knowledge can effectively guide future efforts on the search of promising low GWP alternative refrigerants.

Other molecular-based approaches have also been employed for predicting the thermodynamic behavior of fourth generation refrigerants, such as the series of contributions by Raabe and Maginn^59−65^ on the use of molecular simulations for determining thermodynamic properties of these compounds. Although molecular simulations provide a good detail of physical insights, the required computational time to fully characterize any refrigerant and blends with the required properties for implementing them in cooling applications solely with molecular simulations becomes prohibit expensive. Alternatively, molecular-based EoSs use a coarse-grained approach, preserving the key features of the molecular structure, including their polar nature, with very modest computational requirements. Hence, they have proven to be a successful platform for the assessment of potential low GWP alternative working fluids, primarily due to very limited computational requirements when compared with molecular simulations, while showing excellent extrapolative and predictive power.^54−58^

In this contribution, we present for the first time a robust framework for rapidly assessing the feasibility of replacing single-component third generation refrigerants HFCs with low GWP fourth generation refrigerants HFOs and HCFOs, connecting features of their molecular structure to their performance. The framework is built on the use of a molecular-based EoS, namely, polar soft-SAFT,^66^ for the holistic thermodynamic characterization of the investigated refrigerants. Once the accuracy of the model is established through comparison with available experimental data, the compatibility of third generation refrigerants with their low GWP fourth generation replacements is determined through a drop-in analysis with the objective of minimal retrofitting to the existing system. This analysis is done using technical criteria computed from properties predicted by the thermodynamic model. In addition to these technical criteria, the compatibility of refrigerants is examined in terms of molecular characteristics obtained from the model.

## Methodology

### Selected Technical Criteria for the Drop-in Assessment

Taking third generation refrigerants as benchmarks, it is granted that their fourth generation replacement should satisfy imposed environmental criteria^7^ including low GWP (<150), non- or very low ODP < 0.01, null toxicity (A), and either non- or midflammability (1 or 2 L). These constraints are applicable to all fourth generation refrigerants examined in this work, with the exception of R1243zf, being highly flammable (2), as provided in Table S1 in the Supporting Information (SI).

**Figure 1:**
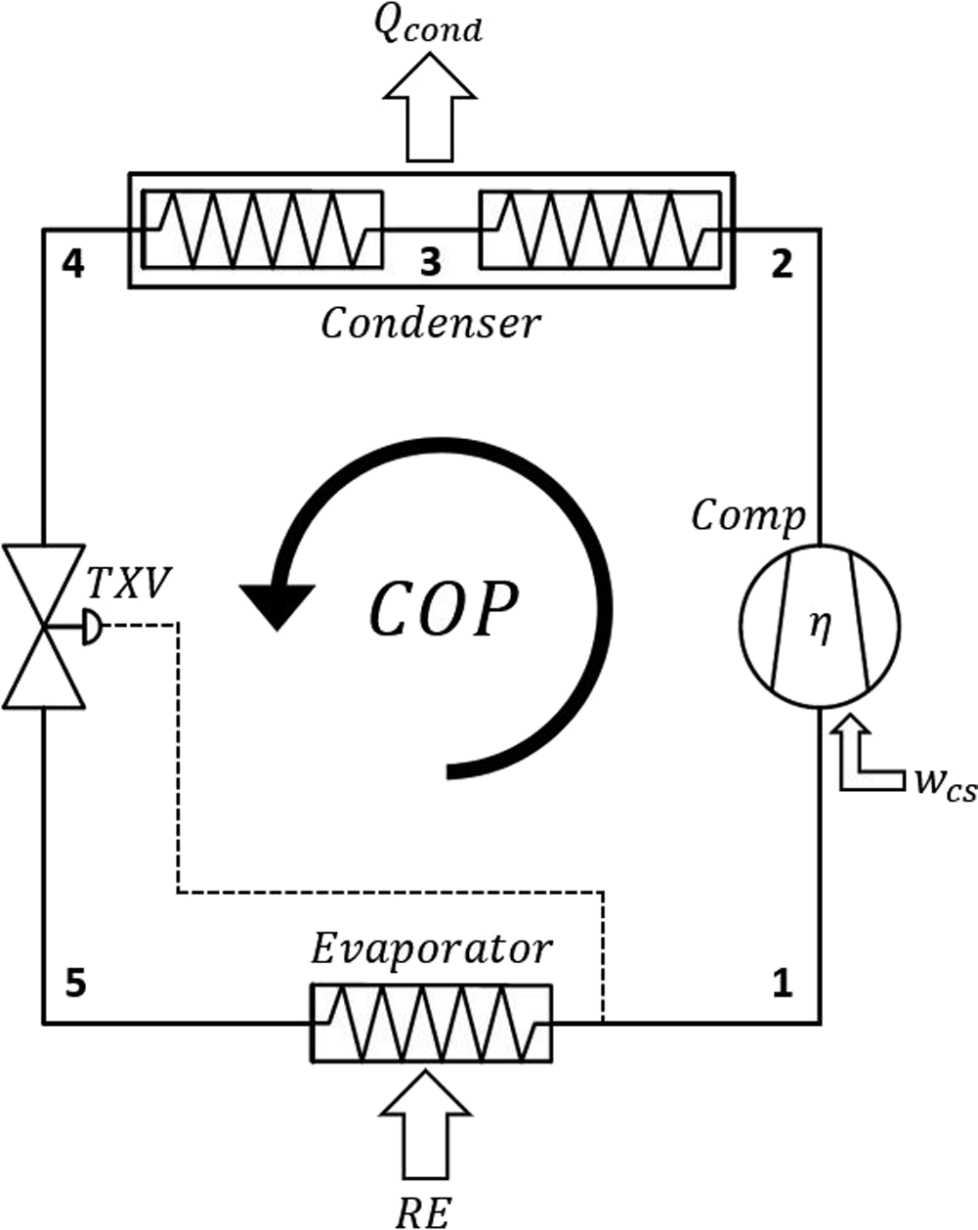
Process flow diagram of the vapor compression refrigeration cycle (VCRC) studied in this work.

Toward assessing the compatibility of fourth generation drop-in replacements, several technical criteria can be included, granted that the technical assessment is done under the same refrigeration cycle and operating conditions. The performance of third generation HFCs and their fourth generation replacements HFOs and HCFOs is assessed in this work using a vapor compression refrigeration cycle as the showcase, displayed in Figure 1. The cooling process is comprised of a single-stage compressor, condenser, evaporator, and thermostatic expansion valve (TXV). The cycle starts with the isentropic compression of the refrigerant (1–2), with the superheated vapor flowing through the condenser, and releasing its sensible (2–3) and latent (3–4) heats to surrounding air to reach the saturation temperature at the condenser’s pressure (2–4). Subsequently, the saturated liquid is expanded resulting in two phase vapor–liquid mixture, with the TXV regulating the refrigerant mass flow rate (4–5), routed to the isobaric evaporator with the refrigerant reaching its saturated vapor phase (5–1). The implementation of the VCRC requires assumptions on the process conditions such as, steady-state flow, fixed evaporator temperature (*T*_evap_ = 270 K), fixed condenser temperature (*T*_cond_ = 300 K), negligible heat transfer in piping, negligible superheating and subcooling effects, zero pressure drop and isobaric conditions in both condenser and evaporator, ideal isentropic efficiency in the compressor, and isenthalpic flow across the TXV, in a manner consistent with our previous work.^67^ These conditions were chosen to simulate a cooling cycle with a medium evaporation temperature (i.e., in the range of 268–283 K)^68^ and a lift temperature of 30 K^69^ to prevent heat cross between external heat sink and the corresponding heat source.^70^

The chosen technical criteria for evaluating compatibility of drop-in replacements include volumetric cooling capacity (VCC), and coefficient of performance (COP). The VCC refers to the amount of cooling per unit volume of the vapor refrigerant at the evaporator outlet, computed as
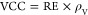
1where RE is the refrigeration effect as the difference between the enthalpies of the refrigerant at evaporator outlet and inlet, while ρ_V_ is the density of the saturated vapor exiting the evaporator, otherwise known as suction density.

Conversely, the COP quantifies the overall efficiency of the refrigeration cycle, expressed as the cooling effect produced per unit of work, as
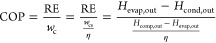
2where *w*_c_ is the actual compressor work, *w*_cs_ is the specific work required by the compressor, and η is the real compressor efficiency, which in this work is assumed to be ideal (i.e., η = 1.0). The computations of these criteria require pressure-enthalpy (PH), and temperature-entropy (TS) diagrams and physicochemical properties predicted using polar soft-SAFT for all studied refrigerants, under the imposed conditions of the cooling cycle.

Refrigerants are deemed compatible if the values of their VCC are similar, denoting equivalent refrigerant volume handled by the compressor without need for compressor retrofitting. Additionally, the replacement refrigerant should possess either a similar or higher COP value compared to the refrigerant to be replaced, denoting either a similar or higher cycle efficiency. For the most compatible refrigerants, other properties with impact on technical performance are examined to gain additional insight on other possible technical trade-offs. These properties include normal boiling point (NBP), condenser pressure (*P*_cond_), suction density (ρ_V_), liquid specific heat (*c_P_*), and refrigeration effect (RE). The normal boiling point measures the ease of vaporizing the refrigerant upon absorbing heat, which should be minimized for low-medium temperature applications, as high NBP would require operating the compressor at low pressures or even vacuum to facilitate vaporization, at the expense of higher operating costs. Similarly, the condenser pressure should also be minimized to reduce the compression power and the operating costs, while the suction density relates to the density of the saturated vapor exiting the evaporator, which should be maximized to reduce the size of the compressor and required capital costs. The specific heat of the refrigerant at liquid state represents the amount of heat required to increase the temperature of the refrigerant by one temperature unit (e.g., 1 *K*), with lower specific heat being preferred as it reduces the amount of heat required to change the temperature of the refrigerant. This is defined as the enthalpy difference of the saturated liquid leaving the condenser at *T*_cond_ (*H*_cond,out_) and the subcooled liquid leaving the condenser at (*T*_cond_ – 1 *K*) (*H*_cond,out_^–1*K*^) at isobaric conditions.

Lastly, the refrigeration effect is a measure of the amount of latent heat absorbed in the evaporator, corresponding to the cooling capacity of the refrigerant, which should be maximized to ensure higher extracted heat or lower refrigerant mass flow rates.

It is important to remark that all systems discussed in this contribution require the use of polyolester (POE)-based lubricant oils, more precisely pentaerythritol esters (PECs), to ensure miscibility, lubricity, and chemical stability of the resulting refrigerant–PEC oil pair. All of the examined drop-in replacements are compatible with PEC; otherwise, a thorough refrigerant–PEC oil compatibility analysis would be required.^67^

### Polar Soft-SAFT Equation

All thermophysical properties of the selected refrigerants considered in this work have been modeled using the polar soft-SAFT Equation of State (EoS),^66^ an extension of the original soft-SAFT equation.^71,72^ Within the framework of polar soft-SAFT,^66^ the residual Helmholtz energy (*a*^res^) of a pure fluid is represented as the sum of various microscopic contributions accounting for its different molecular features, expressed as
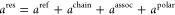
3

The reference term (*a*^ref^) denotes the contribution arising from repulsive and dispersive interactions between individual segments of the reference fluid (a Lennard-Jones (LJ) intermolecular potential). The chain term (*a*^chain^) refers to the contribution resulting from the formation of chains through connectivity of individual segments, while the association term (*a*^assoc^) takes into account the highly directional and short-range interactions such as hydrogen-bonding. Lastly, the polar term (*a*^polar^) explicitly accounts for the contribution from multipolar interactions such as a permanent dipole or quadrupole. The reader is referred to the original contributions for additional details on the expressions of soft-SAFT and its polar extension.^46,47,66,71−73^

The application of polar soft-SAFT to molecular systems entails describing a pure fluid using a coarse-grained representation of its key structural and energetic features, captured through a specific set of molecular parameters. Generally, the model contains a maximum of seven molecular parameters to describe pure fluids, although in most of the cases three or five molecular parameters are used, depending on the governing intermolecular interactions. The most essential parameters required for any pure chain-like fluid include the chain length (*m*_*i*_), LJ segment diameter (σ_*i*_), and LJ segments dispersive energy (ε*_i_*). Two additional parameters are needed to model fluids capable of hydrogen bonding, one related to the volume (κ_α–β, *i*_^HB^) and a second one taking into account the energy of association (ε_α–β, *i*_^HB^). Alternatively, modeling fluids with dipolar or quadrupolar interactions requires, in addition to *m*_*i*_, σ_*i*_, and ε_*i*_, two extra parameters, which are the dipole/quadrupole moment (μ/*Q*), and the fraction of segments affected by the polar moment (*x_p_*). Typically, these polar molecular parameters are either fixed *a priori* based on physical arguments or regressed to the saturated liquid density and vapor pressure of the pure fluid. Hence, for polar compounds, such as those studied in this work, only *m*_*i*_, σ_*i*_, and ε_*i*_ are fitted to experimental data, usually vapor–liquid equilibria.

In this work, a total of 18 different pure refrigerants were studied, provided in Table 1. The studied refrigerants include 10 of the most commonly used single-component third generation refrigerants, along with eight fourth generation refrigerants with demonstrated potential as low GWP alternatives. Although other fourth generation refrigerants have been recently synthesized, some of their environmental and safety properties and thermodynamic properties required for molecular model parametrization are missing, hence, their exclusion from the current work.

**Table 1:**
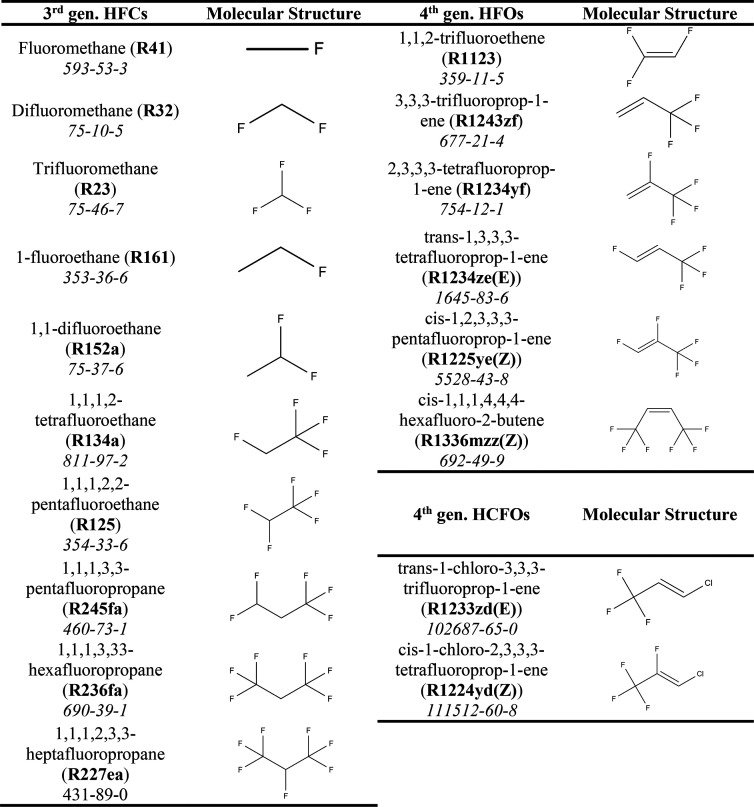
Summary of Refrigerants Modeled in This Work, with Their Commercial Name in Bold and CAS No. in Italic

**Figure 2:**
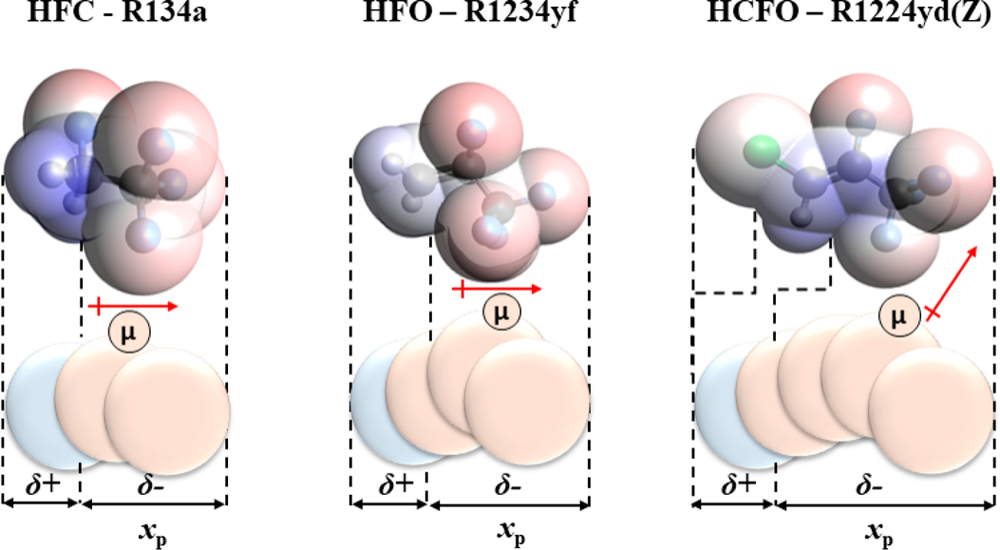
Selected pure refrigerants modeled in this work, with their molecular structure and corresponding polar soft-SAFT coarse-grained molecular model. The beige spheres denote the segments affected by the dipole moment, needed to estimate the fraction of dipolar segments (*x*_*p*_).

The polar soft-SAFT coarse-grained molecular model of these fluids represents them as homonuclear nonassociating LJ chain-like fluids with explicit consideration of their dipole moment, with three representative examples from each family provided in Figure 2. The polar treatment is required due to the presence of halogen atoms (i.e., fluorine, chlorine) in these molecules, leading to the formation of permanent dipole moments arising from the asymmetrical charge distribution. Such explicit treatment is paramount as it ensures high predictive accuracy in modeling pure polar fluid properties and their multicomponent mixtures as demonstrated for other polar fluids in our earlier contributions.^46,47,66,73^ It should be noted that the association term was not included, as the dipolar nature of these refrigerants is far more influential on their thermodynamic behavior.

For the required polar molecular parameters, the dipole moment (μ) was fixed to the experimental value in vacuum, while the fraction of dipolar segments (*x*_p_) was determined *a priori* based on a physical argument taking into account the molecular charge distribution.^46,66^ With this approach, the robustness of the model is preserved, as only three parameters (i.e., σ_*i*_, *m*_*i*_, ε_*i*_) need to be fitted to pure refrigerant experimental saturated liquid density and vapor pressure. Subsequently, these parameters can be used to predict thermodynamic properties required for technical evaluation and even extended to obtain phase equilibria and properties of their multicomponent mixtures. The parametrization of the pure refrigerants has been done using the soft-SAFT proprietary software developed and validated over the past two decades.^74^

## Results and Discussion

### Polar Soft-SAFT Characterization of HFCs, HFOs, and HCFOs

Toward assessing the compatibility of low GWP refrigerants as drop-in replacements, their thermodynamic behavior needs to be characterized, which has been done in this work using polar soft-SAFT EoS. As previously highlighted, the CG molecular models of the examined refrigerants explicitly account for dipolar interactions governing their macroscopic behavior. The optimized polar soft-SAFT parameters for the examined pure refrigerants are provided in Table 2, using the parametrization approach highlighted in the methodology section. Notice that these are nonassociating fluids; hence, *a*^assoc^ in eq 3 is neglected in all calculations.

**Table 2:** Polar Soft-SAFT Molecular Parameters for HFCs, HFOs, and HCFOs Studied in This Work

Compound	*m*	*σ* (Å)	*ε*/*k*_B_ (K)	*μ* × 10^–30^ (C m)^a^	*x*_p_	AAD_P_^b^ (%)	AAD_*ρ*_^c^ (%)	*T* range (K)
**HFCs**								
R41	1.371	3.400	180.3	6.17427	0.50	1.494	0.763	200–280
*R*32	1.376	3.506	164.5	6.59790	0.75	0.565	0.255	200–316
R23	1.397	3.610	147.9	5.50047	0.90	1.327	0.155	200–270
R161	1.577	3.693	232.3	6.47014	0.33	1.374	0.414	200–340
R152a	1.662	3.754	202.3	7.54522	0.50	0.993	0.792	200–356
R134a	1.813	3.770	169.5	6.86475	0.70	1.443	0.389	200–344
R125	1.887	3.790	165.1	5.21360	0.90	1.618	0.274	200–310
R245fa	2.479	3.675	197.1	5.16690	0.80	1.593	0.596	230–395
R236fa	2.056	4.012	172.4	6.61124	0.90	2.410	0.554	270–370
R227ea	2.131	4.033	190.7	4.85669	1.00	4.224	0.260	230–345
**HFOs**								
R1123	1.527	3.760	175.3	5.73730	0.80	2.963	0.199	230–300
R1243zf	1.904	3.880	170.0	8.16890	0.50	1.092	0.295	270–345
R1234yf	1.740	4.082	191.6	6.70790	0.70	1.150	0.314	250–331
R1234ze(E)	2.044	3.821	204.0	4.80330	0.75	3.016	0.555	250–351
R1225ye(Z)	2.077	3.845	172.4	6.03750	0.80	1.018	0.260	250–355
R1336mzz(Z)	1.806	4.430	195.6	10.6406	0.60	3.287	0.565	325–415
**HCFOs**								
R1233zd(E)	2.331	3.819	232.6	3.81190	0.80	2.936	0.506	250–400
R1224yd(Z)	2.278	3.899	202.4	5.63720	0.85	1.358	0.237	280–375

a Experimental dipole moments.^54,61,75−78^

b Experimental vapor pressure from refs (75, 79−84).

c Experimental saturated densities from refs (75, 79, 80, 82, 84−87).

**Figure 3:**
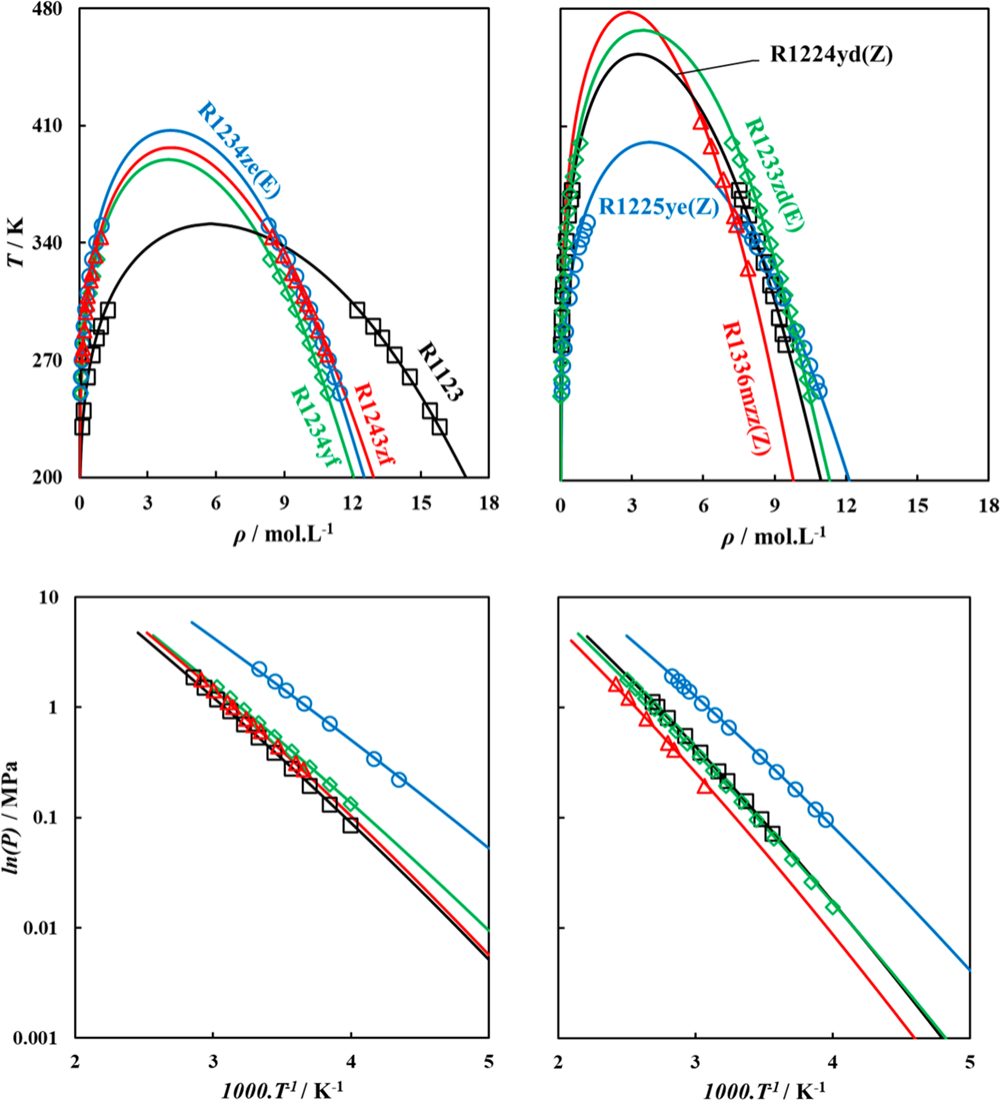
Coexisting densities (top) and vapor pressure (bottom) of pure HFOs and HCFOs studied in this work, with polar soft-SAFT calculations using parameters from Table 2 (solid lines) compared to experimental data (symbols). References for the experimental data are provided in Table 2.

The adequacy of these regressed parameters can be established through comparing polar soft-SAFT calculations for coexisting densities and vapor pressure against experimental data^75,79−87^ used in the parametrization procedure, as provided in Figure 3, for selected fourth generation refrigerants (HFOs and HCFOs). These parameters accurately capture the experimental trends for these properties with an average deviation for saturated liquid density (AAD% ρ_L_^Sat.^*)* below 0.80% in all cases, while for vapor pressure, the average absolute deviation (AAD% *P*^Sat.^) is below 2% in most cases, with some few exceptions that are within an acceptable 4.5%. It should be noted that in the vicinity of the critical region, polar soft-SAFT overestimates the critical properties of the pure fluids, due to the mean-field formulation of the theory. This limitation is shared by all SAFT-based EoSs and can be solved through the addition of the crossover term,^88^ which is outside the scope of this work. Similar model performance was obtained for third generation HFCs, with their polar soft-SAFT computed coexisting densities and vapor pressure included in Figure S1 in the SI

### Effect of Refrigerants Molecular Features on Their Physicochemical Properties

The advantage of the previously optimized parameters is their physical meaning, related to the size and energy of the molecule, allowing extraction of molecular features, and understanding their effect on macrolevel properties. This is of paramount importance as it helps in guiding experimental efforts in the synthesis of alternative refrigerants with desirable physicochemical properties affecting the technical efficacy of the working fluids in their intended applications.

**Figure 4:**
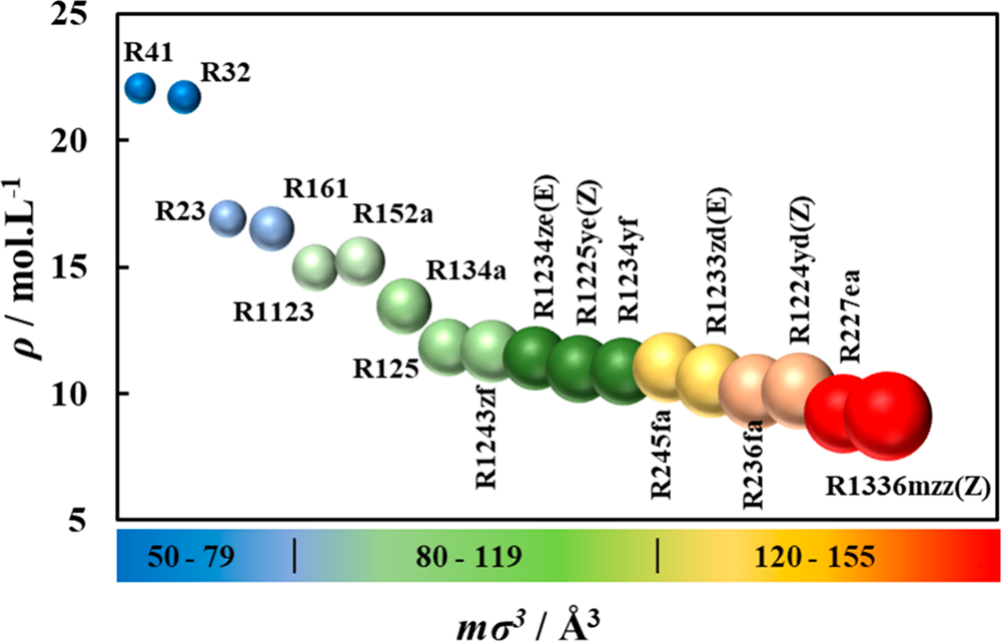
Effect of molecular volume on predicted molar liquid density at *T* = 250 K for refrigerants using polar soft-SAFT. Notice that the size of the sphere reflects the molecular volume as shown in the heat scale.

The change in the liquid density of the studied refrigerants, predicted at *T* = 250 K, is closely related to the volume occupied by the molecule (*mσ*^3^) as provided in Figure 4. The increase in molecular volume results in lower liquid molar densities, which also means higher vapor molar densities. This change is dependent on structural features such as the number of carbons in the chain, number and type of halogenated atoms, and the type of carbon–carbon bonds, with varying degrees of contribution. The most notable structural features with distinct impact on the size of the molecule are the carbon number and degree of halogenation, with their increase leading to increased magnitude for molecular volume, as seen in the case of the single carbon R41, R32, and R23, with increasing fluorine atoms, as well as for increasing number of carbons seen in case of R32 and R152a (both with two fluorine atoms).

Less noticeable effects are associated with the presence of double bonds vs single bonds, the type of halogen atom, and their position in the molecule. The presence of double bonded carbons marginally reduces the molecular size as opposed to single-bonded carbons, due to their lower bond length, as seen for the unsaturated R1225ye(Z) compared to the saturated R245fa. In similar notions, the presence of chlorine marginally increases the molecular size due to its larger atomic radius compared to the fluorine atom, observed for the R1234ze(E) HFO and its counterpart R1233zd(E) HCFO.

On the basis of these trends, and in view of the objective of maximizing the suction density of the refrigerant (vapor phase), it can be expected that larger refrigerants such as R227ea can be an adequate refrigerant judging solely by this criterion, reflective of smaller compressor sizing.

It should be noted that, for practical applications, the analysis can be done on the basis of liquid mass densities, with similar results albeit reversed trends, with refrigerants with lower molecular volumes having lower liquid mass densities consistent with their lower molecular weights.

**Figure 5:**
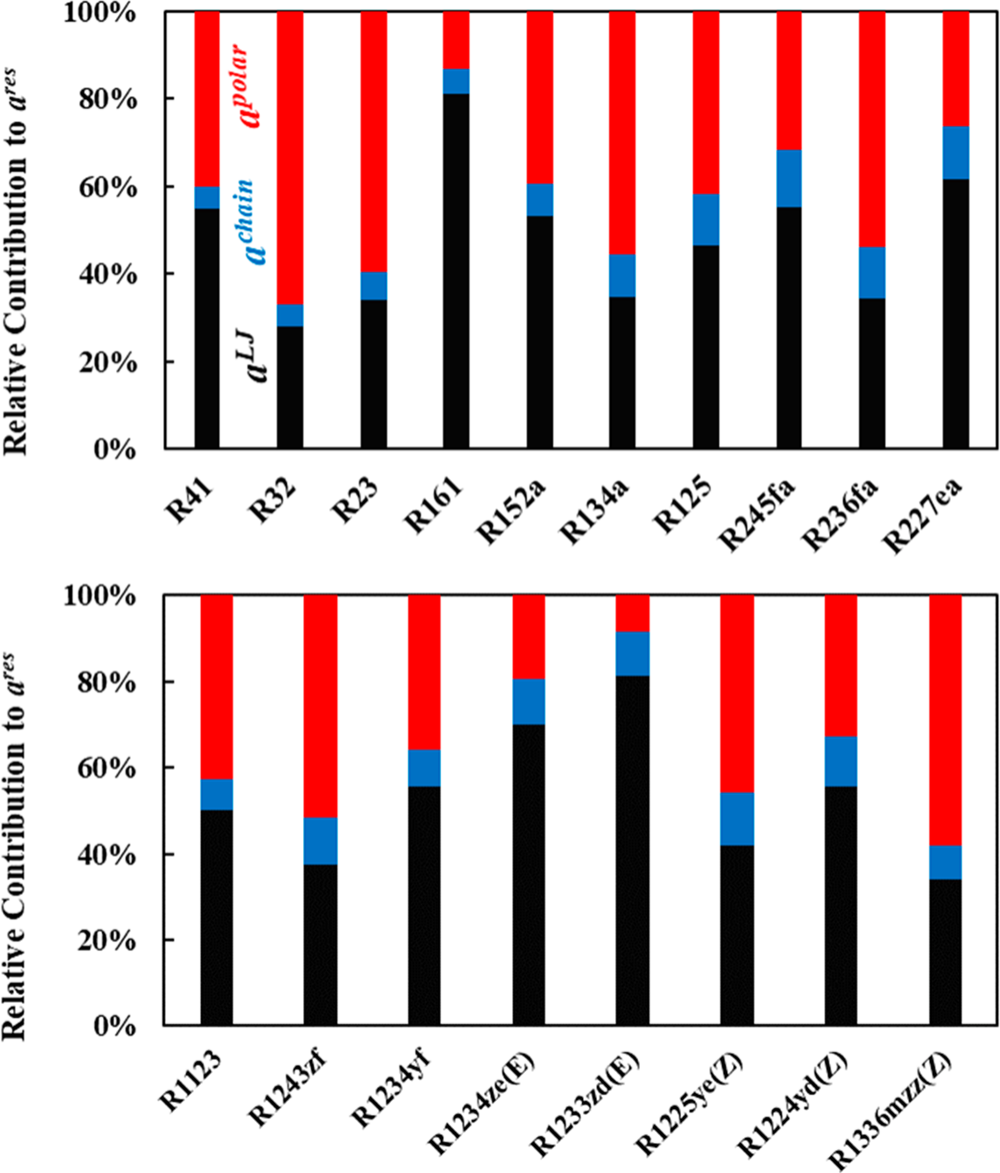
Relative contribution of the various terms (LJ reference, chain, and polar) to the residual Helmholtz energy (*a*^res^) in the CG models for HFCs (top), HFOs, and HCFOs (bottom) predicted from polar soft-SAFT at *T* = 250 K and *P* = 0.1 MPa.

To better elucidate the importance of explicitly accounting for the polarity of the refrigerants investigated herein, the relative contribution of the different terms (i.e., LJ reference, chain, and polar) to the residual Helmholtz free energy (eq 3) were predicted from the thermodynamic model at *T* = 250 K and *P* = 0.1 MPa, as provided in Figure 5.

For the majority of the refrigerants, the polar contribution to the Helmholtz energy has a large impact in the range of 25%–67%, with the exception for R1233zd(E) and R161, clearly demonstrating the importance of explicitly including the effect of dipolar interactions in their CG molecular models. These predictions can help in understanding the relative importance of the different intermolecular interactions on the physicochemical properties, similar to the aforementioned case with density. For saturated HFCs with similar carbon number such as R161, R152a, R134a, and R125 (all with two carbon atoms), the increasing degree of fluorination is accompanied with increased contribution of dipolar interactions compared to dispersive, provided in Figure 5 (top). This is distinctly seen moving from R161 (1 F) and R152a (2 F) to R134a (4 F), associated with the assumed increase in molecular segments influenced by the dipole moment (*mx*_p_). Notice that the move from R134a (4 F) to R125 (5 F) resulted in a reduction in the polar contribution attributed to the reduced dipole moment of the latter owing to the effect of the additional fluorine atom on making the charge distribution more symmetrical. The same trends were also observed for HFCs with one carbon atom (i.e., R41, R32, and R23), and three carbon atoms (i.e., R245fa, R236fa, and R227ea).

Shifting the focus on the effect of the carbon number with similar degrees of halogenation, R41 vs R161 (1 fluorine atom) and R32 vs R152a (two fluorine atoms), it is observed that the polar contribution decreases with increasing carbon chain length, as the increased size of the molecule (Figure 4) reduces the portion of the molecule influenced by the dipole moment, irrespective of its magnitude. This behavior has also been previously observed for the 2-ketones family.^66^

The effect of the degree of fluorination is quite different in the case of unsaturated HFOs and HCFOs, shown in Figure 5. For HFOs with a similar carbon number (i.e., three carbons) such as R1243zf, R1234yf, R1234ze(E), and R1233zd(E), the polar contribution is reduced with increasing the degree of fluorination. This can be associated with the higher polarizability of the double bonded carbons compared to single-bonded carbons, leading to a more symmetrical charge distribution, dampening the effect of the dipole moment. The type of halogen atom also has an influence on the polar contribution, with HCFOs having a lower impact compared to their HFOs counterparts (i.e., R1233zd(E) vs R1234ze(E)), consistent with the lower electronegativity of chlorine compared to fluorine. Also, notice the effect of the position of fluorine atoms seen in the case of R1234yf vs R1234ze(E), with the increased polar contribution of R1234yf due to the proximity of the isolated fluorine to the fully fluorinated carbon, increasing the overlapping electrostatic potential and, consequently, the polarity of the molecule.

These molecular tendencies will manifest on properties such as vapor pressure and enthalpy of vaporization. For example, the low vapor pressure for R1336mzz(Z) is due to its high dipole moment resulting in the highest polar contribution (Figure 3). Still, it remains quite difficult to distinctly isolate the effect of polar interactions on these properties as they are affected by the combined contribution of structural effects and intermolecular interactions. Note that the contributions reported in Figure 5 are relative rather than absolute, normalized to each refrigerant’s residual energy.

### Validation of Refrigerant Coarse-Grained Molecular Models

The strength of using molecular-based EoSs, even if some limited experimental data are needed for parametrization, is that once the CG molecular models are developed, they can be used in a fully predictive manner to obtain other thermodynamic properties not included in the parametrization. These predictions typically serve as another layer of reliability and accuracy testing, especially in the case of first and second order derivative properties, due to their heightened sensitivity to errors in modeling the vapor–liquid equilibria (VLE) of the pure fluid.^89,90^ Additionally, explicit inclusion of dipole–dipole interactions in the model is paramount for accurate predictions of these derivative properties, as demonstrated in other contributions.^48,66^

**Figure 6:**
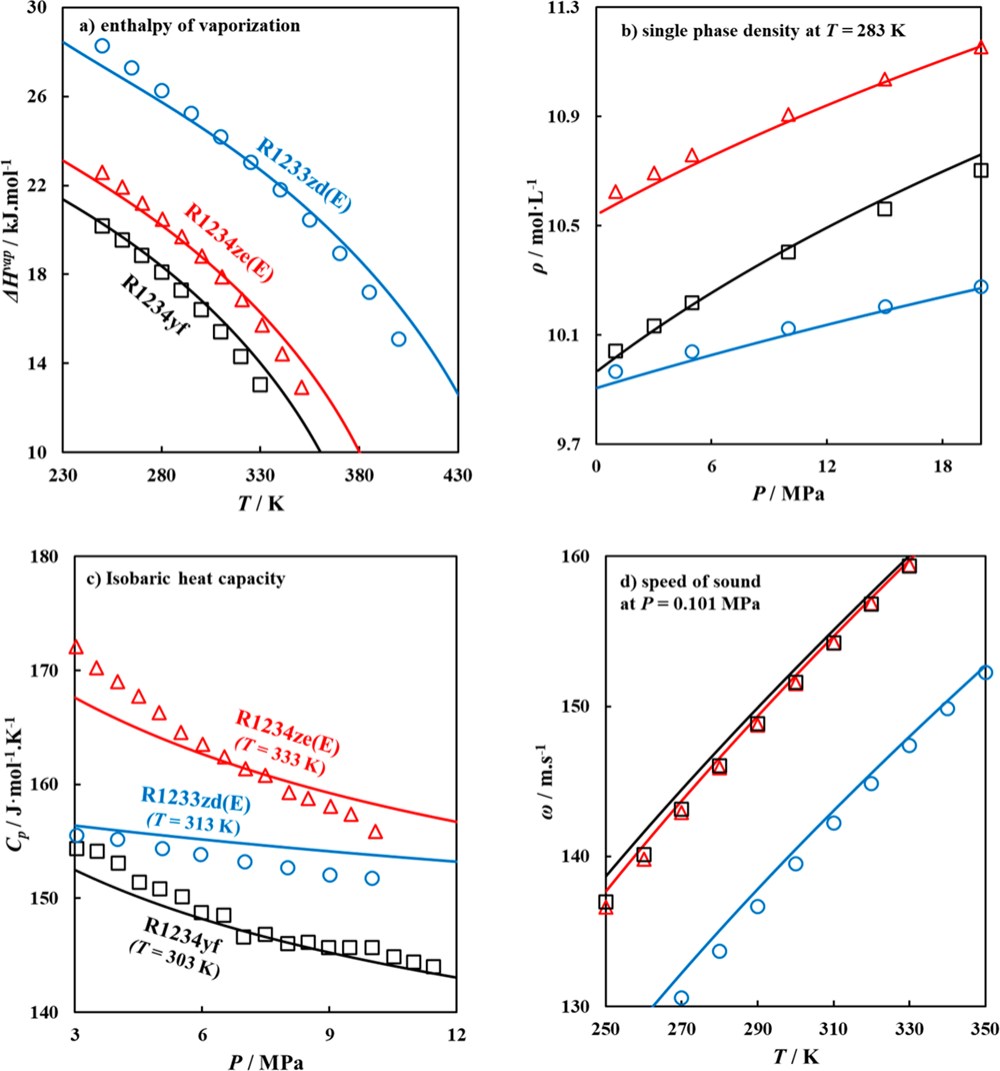
Thermodynamic properties of low GWP refrigerants, including (a) enthalpy of vaporization, (b) single-phase density, (c) isobaric heat capacity, and (d) speed of sound, for R1234ze(E), R1234yf, and R1233zd(E), with polar soft-SAFT predictions (solid lines) compared to experimental data^75,92−94^ (symbols).

The additional predicted properties for the modeled refrigerants, include enthalpy of vaporization (*ΔH*^vap^), single-phase density, isobaric heat capacity (*C*_P_), and speed of sound (ω), as provided in Figure 6, for selected fourth generation refrigerants, R1234yf, R1234ze(E), and R1233zd(E), while those for the remaining refrigerants can be found in Figures S2–S5 in the SI. It should be noted that predictions for the isobaric heat capacity require the inclusion of the ideal gas isobaric heat capacities (*C*_p_^IG^), obtained from available literature data.^75,91^ The agreement between polar soft-SAFT predictions and experimental data^75,92−94^ is excellent (AAD = 1.5%) for enthalpy of vaporization, single-phase density, and speed of sound, while the deviations for predicted isobaric heat capacity are within an acceptable 5%, further attesting the accuracy and predictive capability of the equation in modeling refrigerants. The enthalpy of vaporization is another measure for the strength of the intermolecular interactions, with higher values reflective of stronger intermolecular interactions, as seen in the case of R1233zd(E), with its larger dipole moment and higher dipolar molecular segments compared to the others, requiring more energy to transition the molecule from liquid to vapor phase. Additionally, the change in single-phase densities for the three refrigerants is consistent with their molecular volumes (Figure 4), with R1233zd(E) having the lowest single-phase density due to its larger molecular volume compared to the former two.

**Figure 7:**
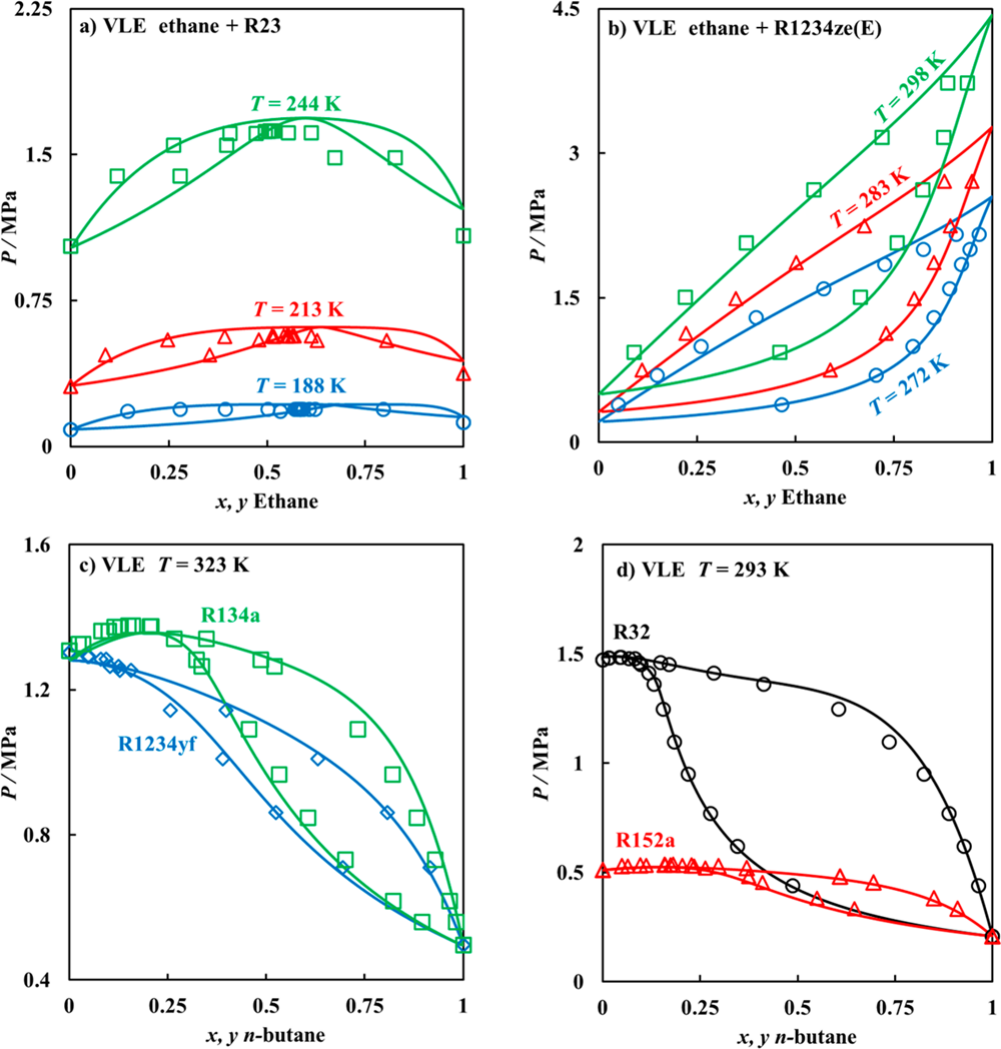
Predicted VLE for binary mixtures of (a) ethane + R23, (b) ethane + R1234ze(E), (c) *n*-butane + R134a or R1234yf at *T* = 323 K, and (d) *n*-butane + R32 or R152a at *T* = 293 K. In all cases, polar soft-SAFT predictions (solid lines) are compared to experimental data^95−100^ (symbols).

The last demonstration of the accuracy of the model in capturing the effect of dipolar interactions is demonstrated through predicting the VLE for binary mixtures of dipolar + nonpolar fluids. In this manner, the ability of the model to correctly approximate the contribution of dipole–dipole interactions can be isolated, as dipole interactions have a larger effect on the nonideality of their VLEs with nonpolar fluids.^46,47^ For this end, binary mixtures of dipolar refrigerants with nonpolar *n*-alkanes (modeled as a chain-like LJ fluid, using parameters included in Table S2 in the SI)^74^ were predicted from the thermodynamic model for mixtures including ethane with R23 or R1234ze(E) and n-butane with R32, R152a, R134a, and R1234yf as dictated by available experimental data.^95−100^ Indeed, the agreement between the experimental VLE data and model predictions is excellent with AADs = 1.0%, as seen in Figure 7, merely from the accurate representation of the behavior of the pure refrigerants.

These behaviors can also be better explained from the microlevel insights provided in Figure 5. For binary mixtures with ethane, the prominent polar contribution of R23 resulted in the formation of a distinct azeotropic mixture at nearly equimolar concentrations, while the lower polar contribution of R1234ze(E) due to its larger molecular volume resulted in an almost ideal VLE behavior. Conversely, the formation of azeotropes at high refrigerant concentrations was seen for all binary mixtures with *n*-butane, with the degree of nonideality associated with the magnitude of the polar contributions of the refrigerant. For saturated R134a, its high polarity compared to R1234yf resulted in a more distinct positive azeotrope, due to the increased asymmetrical energy scale between the stronger polarity of R134a with the nonpolar *n*-butane. Notice, both refrigerants possess relatively similar dipole moments and fraction of polar segments; however, the larger molecular volume of R1234yf contributes to reducing its overall dipolar interactions. A similar observation is seen in the case of R32 and R152a, with a higher deviation from nonideal behavior for mixture with R32 manifested in the form of a larger spread for the VLE. Even though the magnitude of the dipole moment of R152a is higher than R32, still its larger size reduces the portion of the molecule influenced by the dipole moment.

### Drop-In Assessment for Low GWP Refrigerants

In this section, we present results on the drop-in assessment for the most compatible low GWP fourth generation refrigerants as replacements for the currently used third generation refrigerants. The assessment is done employing the technical criteria highlighted in the methodology for the examined VCRC systems. The PH- and TS-diagrams for all the pure refrigerants as predicted from polar soft-SAFT EoS, under the imposed operating conditions for the VCRC, for all the examined refrigerants are shown in Figures S6 and S7 in the SI. The values for the ideal gas enthalpy (*H*^IG^), and entropy (*S*^IG^) required for these predictions from the thermodynamic model were taken from literature.^75^ Moreover, all other physicochemical properties needed to complete the assessment were also obtained from polar soft-SAFT in a predictive manner.

The most convenient criterion for analyzing compatibility of drop-in replacements would be merely using VCC under the same VCRC operating conditions, as currently implemented in most works on searching for alternative refrigerants.^83,101,102^ Similar VCC values between benchmark third generation refrigerant, and low GWP fourth generation replacement signifies their compatibility without the need to retrofit units in existing refrigeration cycle, in particular the compressor.^103^

**Figure 8:**
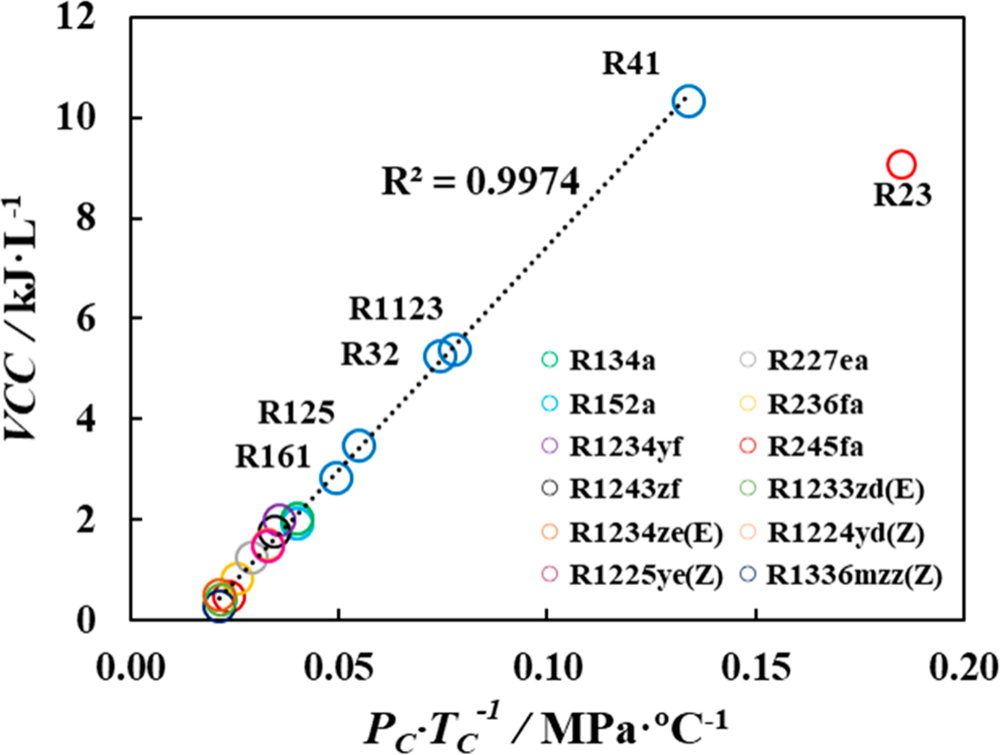
Correlation for polar soft-SAFT predicted VCC values of refrigerants examined in this work and their experimental critical properties from the literature.^75,81−83,105−108^

Prior to implementing this criterion, it is worthwhile to test the validity of VCC predictions using polar soft-SAFT EoS (eq 1). This is done through linearly correlating predicted VCC values with the critical properties (i.e., critical temperature and pressure) of the pure refrigerants,^104^ indicating the accuracy of the predicted VCC values. The critical properties for each refrigerant were obtained from the literature,^75,81−83,105−108^ while VCC were directly predicted from polar soft-SAFT. As provided in Figure 8, the predicted VCC values and critical properties of the studied refrigerants indeed followed a linear correlation (*R*^*2*^ > 0.997), with the exception of R23. This outlier was expected as the critical temperature of R23 (*T*_c_ = 299.29 K) is very close to the condenser operating temperature for the VCRC (*T*_cond_ = 300 K). It should be noted that a refrigerant with a low-critical temperature such as R23 most likely would require the use of a transcritical cycle if operated at medium-temperature conditions.^109^ Notwithstanding, this simple test affirms the accuracy and predictive power of polar soft-SAFT in capturing the behavior of key technical criterion for evaluating the compatibility of drop-in replacements.

**Figure 9:**
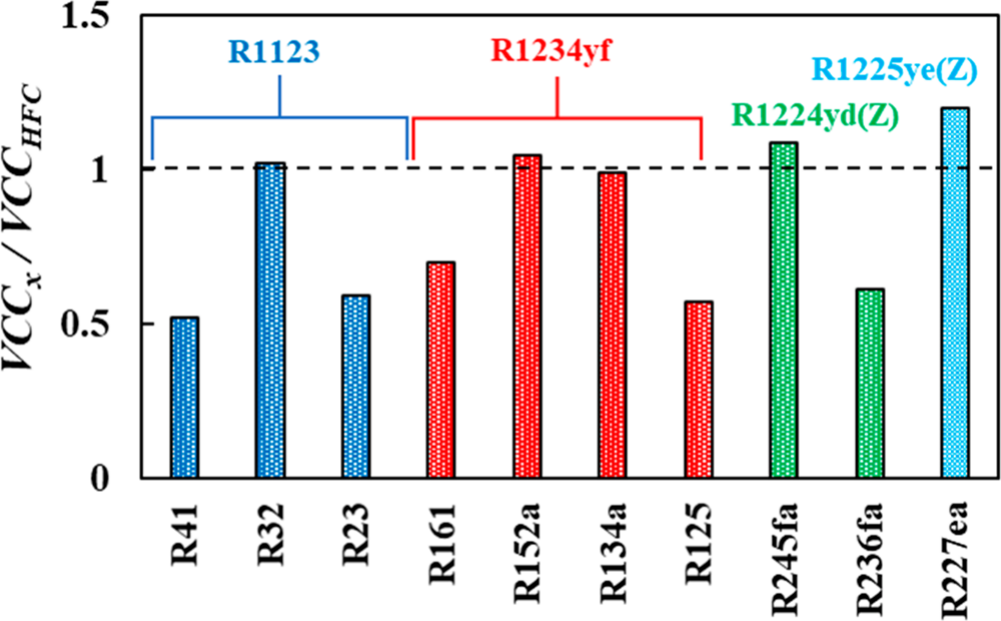
Compatibility test for replacing third generation refrigerants with fourth generation refrigerants based on polar soft-SAFT predicted VCC shown in Figure 8. The dashed line denotes similar VCC for third generation benchmark and its fourth generation drop-in replacement.

Highlighted in Figure 9 are the most compatible fourth generation refrigerants with the 10 third generation single-component refrigerants (HFCs) as benchmarks. It can be seen that for some distinct cases, such as those for replacing R32, R152a, R134a, and R245fa, fourth generation refrigerants are nearly compatible as drop-in replacements using working fluids such as R1123, R1234yf, and R1224yd(Z), with their VCC values being within 10% of those for the benchmark refrigerants to be replaced. Conversely, other refrigerants are available, such as replacing R41 or R23 with R1123; however, the low VCC value of R1123 indicates that the refrigeration cycle needs to be retrofitted. These dissimilar VCC values require an increase in the compressor size for equivalent volumes of compressor-displaced refrigerant. In this aspect, rational design of refrigerant blends might prove efficient for the replacement of those refrigerants without compatible low GWP single refrigerants. This assessment has also been done at different evaporator and condenser temperatures to evaluate their impact, as included in Figure S8 in the SI, yielding relatively similar outcomes to the most compatible fourth generation drop-in replacements. Notice the effect of increasing the lift temperature on decreasing the corresponding VCC values.

On the basis of the previous compatibility assessment, we further investigate drop-in replacements for R32, R152a, R134a, and R245fa, inclusive of other technical criteria often overlooked. This is done through estimating the corresponding trade-off between COP versus VCC as well as a thorough review of their main thermophysical properties. The drop-in assessment is performed considering potential drop-in replacements with VCC and COP values within 25% of the reference refrigerant to be replaced. It should be mentioned that for R1336mzz(Z) and R1224yd(Z), the COP is predicted as an average based on available experimental data under similar conditions,^110,111^ due to the nonavailability of isothermal ideal gas entropies needed to compute the TS-diagrams involved in the COP calculations. The COP values for these refrigerants are within those obtained for highly efficient refrigerants R1233zd(E) and R245fa. Moreover, the outcome of this evaluation has been done at arbitrary conditions, which may not be optimal for each refrigerant. However, the results established the benefit associated with the evaluation platform developed in this work for the rapid assessment of drop-in replacement refrigerants, allowing a more targeted evaluation and optimization for the most promising ones.

**Figure 10:**
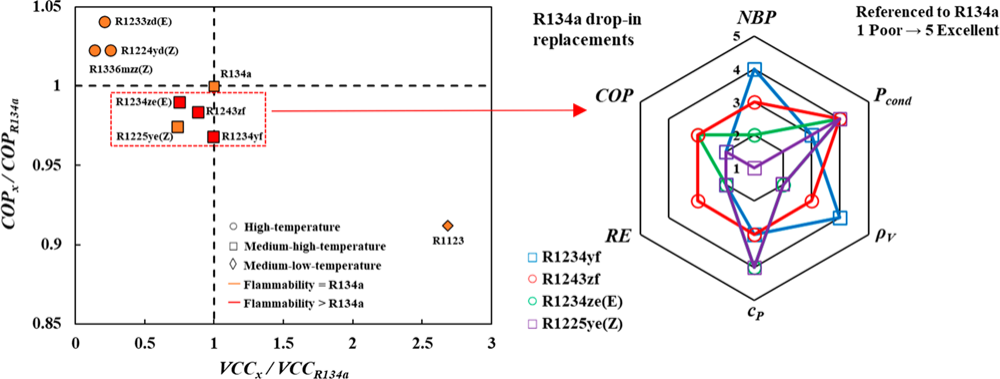
Drop-in analysis for replacing R134a based on VCC and COP benchmarked to R134a (left), including other technical criteria relative to R134a for promising replacements (right). See text for details.

The initial focus for the detailed drop-in analysis was on R134a, shown in Figure 10, being the most widely used working fluid in a diverse range of applications such as domestic refrigerators, commercial chillers, stationary refrigeration equipment, and medium temperature mobile air conditioning systems, with the urgent need to find a suitable replacement.^6,7,112,113^ Among the examined possible replacements, R1234yf and R1243zf, as well as R1234ze(E) and R1225ye(Z) to a lesser extent, emerged as attractive options solely based on their technical performance with nearly similar VCC values, albeit more reduced COP. This might demonstrate that their usage has the possibility of not incurring additional capital cost associated with altering the design of the compressor but on the expense of reduced system efficiency, which can be acceptable (performance drop inferior to 3.2% in all cases) considering their significantly lower GWP. Judging by additional technical criteria, R1234yf is indeed an attractive technical option with the limited need for adjusting the design of the compressor as supported by its NBP, its suction density being higher than that for R134a, and relatively similar condenser operating pressure and liquid specific heat. Briefly, it might seem that the incurred costs might be mainly operational due to the higher energy demand for compression, as demonstrated from its lower RE. On another front, especially with the inclusion of the flammability of these options, it is established that R1225ye(Z) might prove a better option owing to its safer operation due to its nonflammable nature (similar to that of R134a) as opposed to the mildly flammable R1234yf (though more technically compatible) or the highly flammable R1243zf, which would be reflected on the cost needed for the installation of additional safety layers. This exemplifies the difficulty in finding a single-component refrigerant encompassing environmental/safety requirements and technical performance.

Recent publications^32,114^ on searching for replacements for R134a (GWP = 1300)^115^ indicated the promising use of the third generation refrigerant R152a (GWP = 138)^115^ for oil-free automotive air conditioning, guided by its lower GWP, low cost, and minimal equipment change. Nonetheless, its highly flammable nature is a concern; hence, based on the outcome of the analysis in this work, R1225ye(Z) (GWP = 3^115^ and with nonflammability 1) is a more competent alternative to replace R134a, as opposed to R152a. The required modifications might include enlarging the compressor rotation speed or use of an internal heat exchanger (IHX) to ensure similar operation to cycles using R134a.^116^ Other hardware modifications can be included such as tuning the TXV (Figure 1) and setting up a variable displacement compressor control valves, which cannot be exceedingly expensive if compared to a complete system redesign.^26^ It is worth remarking that for drop-in replacements with higher flammability such as the case for R1234yf, safe operation can be ensured through reducing the refrigerant load in the cooling cycle.^117^

**Figure 11:**
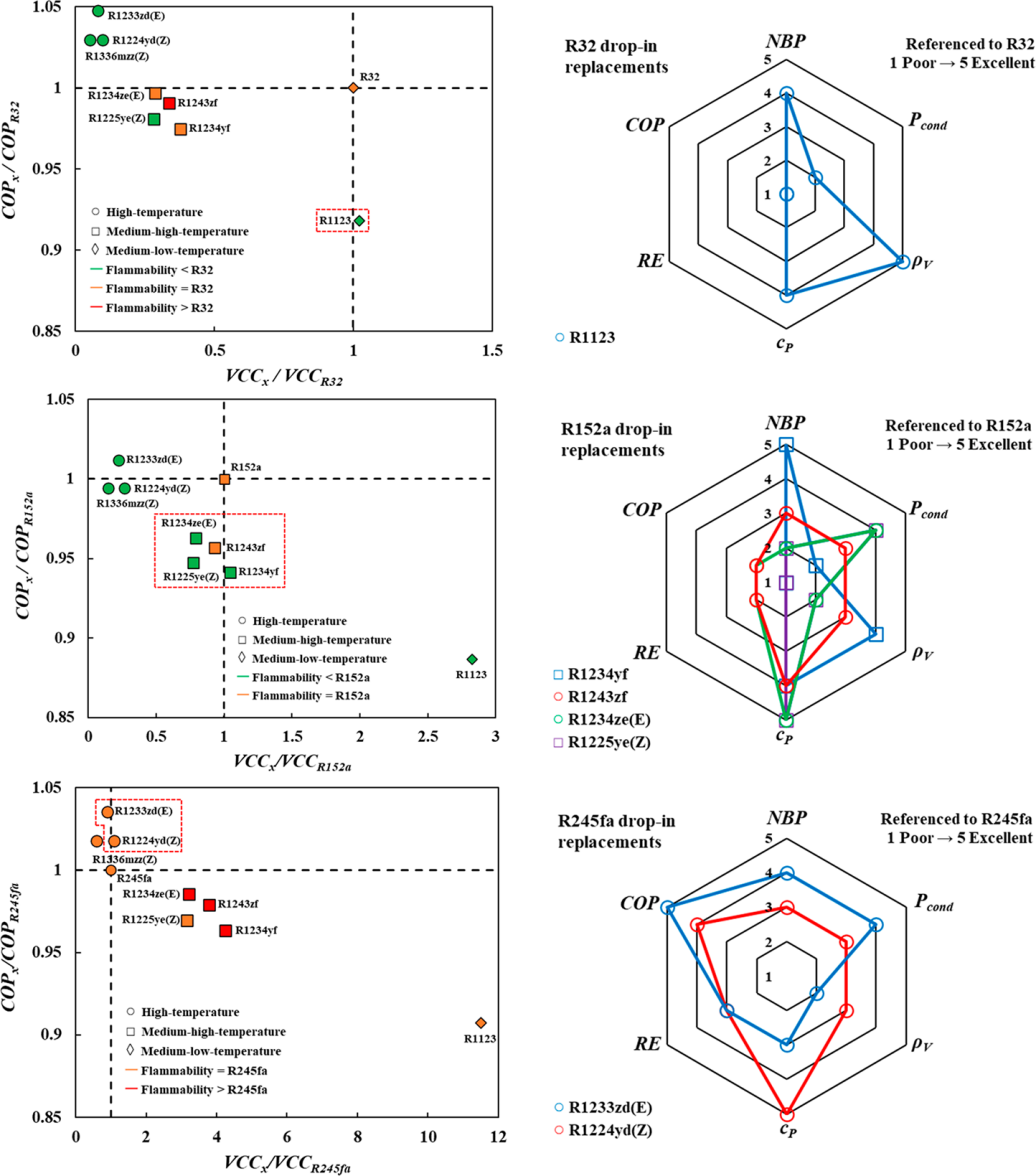
Drop-in analysis for replacing R32, R152a, and R245fa with fourth generation refrigerants, based on VCC and COP benchmarked to each refrigerant (left), including other technical criteria relative to each refrigerant (right). See text for details.

In line with the previous analysis, the ability of drop-in replacements for other third generation refrigerants R32, R152a, and R245fa is included in Figure 11, to demonstrate the flexibility of the approach proposed in this work. In the current market, R32 (GWP = 677)^115^ is used as a medium-GWP drop-in replacement to R410A (GWP = 2088),^115^ which will also be phased out soon. The most compatible drop-in replacement for R32 proved to be R1123, with a relatively similar VCC value, albeit an 8.2% reduction in its COP. Although other fourth generation refrigerants have similar COPs to R32, their significantly lower VCCs indicated the need to fully retrofitting systems operating with R32. Notwithstanding, the prospect analysis of using R1123 as a drop-in replacement for R32 in terms of other KPIs showed that the only concerning factors would be the higher condenser pressure and lower RE of R1123, translated to increased operating costs. Moreover, the nonflammability of R1123 would not require any adjustments in the pre-existing risk control measures.

The working fluid R152a is currently deployed in cascade refrigeration configurations with transcritical CO_2_; however, even though its GWP is within acceptable environmental requirements, its high flammability is a concern, requiring its immediate replacement.^118−120^ In the case of drop-in replacement for R152a, several options are available including R1234yf, R1243zf, R1234ze(E), and R1225ye(Z), with comparable VCCs and nearly 6.0% reduction in COPs. Although R1234yf seemed like a good option judging solely on its VCC and safety concerns, other technical criteria showed a reduced performance compared to R152a. On the other hand, a better compromise on COP, liquid specific heat, and condenser pressure is achieved when employing R1234ze(E) and R1225ye(Z) as drop-in replacements.

Lastly, R245fa is considered as the reference working fluid for high-temperature heat pumps and organic Rankine cycles.^121^ For replacing R245fa, high-NBP fourth generation refrigerants such as R1224yd(Z) and R1233zd(E) were found to be appropriate, along with demonstrated enhanced COP. These refrigerants are more applicable to high-temperature air condition applications or systems operating under low-pressure centrifugal configurations, due to their similar saturated vapor pressures and critical properties, promising high efficiency and reduced VCCs.^25,109^ The most compatible option would be R1224yd(Z) owing to its improved COP and suction density resulting in enhanced cooling cycle efficiency, while reducing the mass flow rate of the working fluid in the compressor line. Moreover, R1224yd(Z) as a drop-in replacement maintains relatively similar VCC, condenser pressure, RE, and normal boiling point, without incurring any costs for system retrofitting. Larger improvements are gained with this drop-in replacement owing to its low GWP, along with its nonflammable nature and lower toxicity. Similar findings were demonstrated by Mateu-Royo et al.^101^ reinforcing the potentiality of R1224yd(Z) as an attractive replacements for R245fa. Notice that for R245fa drop-in assessment, the NBP performance is computed with reference to its maximization owing to its use in high-temperature applications, as opposed to the low-medium temperature application of the aforementioned cases when finding replacements for R134a, R32 and R152a.

All these possible alternatives can be rationalized in view of their microlevel features. Notice that the replacements of a third generation with a fourth generation refrigerant is possible for those with similar structural characteristics such as carbon number and degree of fluorination. For example, compatibility of R134a and R152a with R1234yf is due to them being within the same molecular space in terms of carbon number and degree of fluorination, with a fine balance between structure and energy affecting the thermodynamic properties. Their similar VCC values relative to R134a or R152a are due to the reduced vapor density of the replacements (owing to their slightly larger molecular volume), compensated with their increased enthalpy due to their higher polarity. Shifting farther from these structural features, such as seen in the case of the larger and less polar HCFOs such as R1224yd(Z) and R1233zd(E), skewed the fine balance of molecular features on their properties compatible with R134a or R152a. Furthermore, they proved to be compatible with R245fa due to similar structural characteristics.

The outcome of this analysis clearly demonstrates not only the power of polar soft-SAFT as a robust and predictive thermodynamic model enabling the systematic evaluation of next generation refrigerants but also its capability of providing molecular level insight on the influence of structure and interactions on the compatibility of drop-in replacements. As such, future efforts on the synthesis of alternative replacements should build on the molecular features of the refrigerant to be replaced, by ensuring that the replacement maintains relatively similar features within the same molecular space.

## Conclusions

In this work, polar soft-SAFT molecular-based equation of state was used as a platform to rapidly assess the suitability of fourth generation refrigerants over their third generation counterparts from their technical performance, taking into account the influence of their molecular structure on their performance, while also considering their compatibility with current environmental legislations. The evaluation framework accounted for an extensive number of technical criteria for the utilization of these low GWP refrigerants as drop-in replacements in a vapor compression refrigeration cycle, as a showcase. Toward the implementation of the technical evaluation, the polar soft-SAFT EoS established itself as a crucial thermodynamic model by fully characterizing thermodynamic properties of 18 different third and fourth generation refrigerants. The explicit inclusion of the dipolar interactions of these refrigerants ensured a highly accurate and robust model as demonstrated from predicted first and second order thermodynamic derivative properties of the refrigerants, along with accurate representation of binary mixtures of refrigerants with *n*-alkanes. The physical basis of the model enabled the extraction of molecular characteristics of the studied refrigerants and their effect on the physicochemical properties impacting their technical efficacy.

The subsequent drop-in assessment established that R32, R152a, R134a, and R245fa, some of the most commonly commercialized working fluids, could be potentially replaced with lower GWP fourth generation refrigerants, without incurring on additional capital cost associated with compressor modifications. The most viable replacements included R1123, R1225ye(Z), R1234ze(E), and R1224yd(Z); however, the expected downfall might be the additional operating costs associated with required compression energy. After establishing the relationship between their molecular features and their performance, it can be concluded that the compatibilities of these replacements with their predecessors are attributed to their relatively similar molecular features. Additionally, for the remaining third generation refrigerants (R41, R23, R161, R125, R236fa, R227ea), although no suitable replacement was found, rational design of blended refrigerants might prove feasible for their replacement, entailing a detailed blend analysis expected in future contributions.

The collective results presented herein not only demonstrate the ability of the proposed approach, built on molecular modeling, but also assists in rapidly examining the inherent trade-offs or possible issues in selecting next generation sustainable drop-in replacements. It should be noted that an important factor remains missing in this drop-in analysis, which is the cost of the replacement refrigerants. However, as the current market is shifting toward deploying alternative compounds in line with environmental laws, it can be expected that the production costs of these emerging refrigerants will become cheaper with increased productivity.

## Supporting Information Available

Figures on vapor pressure, coexisting densities, heat capacity, enthalpy of vaporization, and pressure-enthalpy and temperature-entropy diagrams obtained from polar soft-SAFT EoS for the selected refrigerants modeled in this work (PDF)
