# Supplementary material for: Assessment of Low Global Warming Potential Refrigerants for Drop-In Replacement by Connecting their Molecular Features to Their Performance
Source: ACS Sustain Chem Eng. 2021 Dec 7;9(50):17034–48. doi: 10.1021/acssuschemeng.1c05985 (PMC8693772; doi:10.1021/acssuschemeng.1c05985)
Supplement: Supplementary file 1 — sc1c05985_si_001.pdf [file sc1c05985_si_001.pdf]

# Supporting Information

# Assessment of low Global Warming Potential Refrigerants for drop-in Replacement by Connecting their Molecular Features to their Performance

Carlos G. Albà[†‡], Ismail I.I. Alkhatib[†], Fèlix Llovell[‡*], Lourdes F. Vega[†*]

[†]Research and Innovation Center on $CO_2$ and $H_2$ (RICH), Chemical Engineering Department, Khalifa University, PO Box 127788, Abu Dhabi, United Arab Emirates.
[‡]Department of Chemical Engineering, ETSEQ, Universitat Rovira i Virgili (URV), Av. Països Catalans 26, 43007, Tarragona, Spain.

Corresponding authors e-mail addresses:

Lourdes F. Vega: lourdes.vega@ku.ac.ae

Fèlix Llovell: felix.llovell@urv.cat

Number of figures: 8 (Figures S1-S8)

Number of tables: 2 (Table S1-S2)

Number of pages: 9 (S1-S9)

**Table S1.** Environmental, and Safety for refrigerants examined in this work included in **Table 1**.[1–8]

| Substance | GWP | ODP | Toxicity | Flammability |
|---|---|---|---|---|
| R41 | 116 | 0 | A | 2 |
| R32 | 677 | 0 | A | 2L |
| R23 | 12400 | 0 | A | 1 |
| R161 | 4 | 0 | A | 2 |
| R152a | 138 | 0 | A | 2 |
| R134a | 1300 | 0 | A | 1 |
| R125 | 3170 | 0 | A | 1 |
| R245fa | 858 | 0 | B | 1 |
| R236fa | 8060 | 0 | A | 1 |
| R227ea | 3350 | 0 | A | 1 |
| R1123 | 3 | 0 | A | 1 |
| R1243zf | 1 | 0 | A | 2 |
| R1234yf | 0 | 0 | A | 2L |
| R1234ze(E) | 1 | 0 | A | 2L |
| R1225ye(Z) | 3 | 0 | A | 1 |
| R1336mzz(Z) | 2 | 0 | A | 1 |
| R1233zd(E) | 5 | 0.00024 | A | 1 |
| R1224yd(Z) | 1 | 0.00023 | A | 1 |

**Table S2.** Soft-SAFT molecular parameters of *n*-alkanes used in this work, as transferred from previous work.[9]

| Substance | $m$ | $\sigma$ (Å) | $\varepsilon/k_B$ (K) |
|---|---|---|---|
| Ethane | 1.392 | 3.728 | 147.2 |
| *n*-butane | 2.134 | 3.871 | 237.7 |

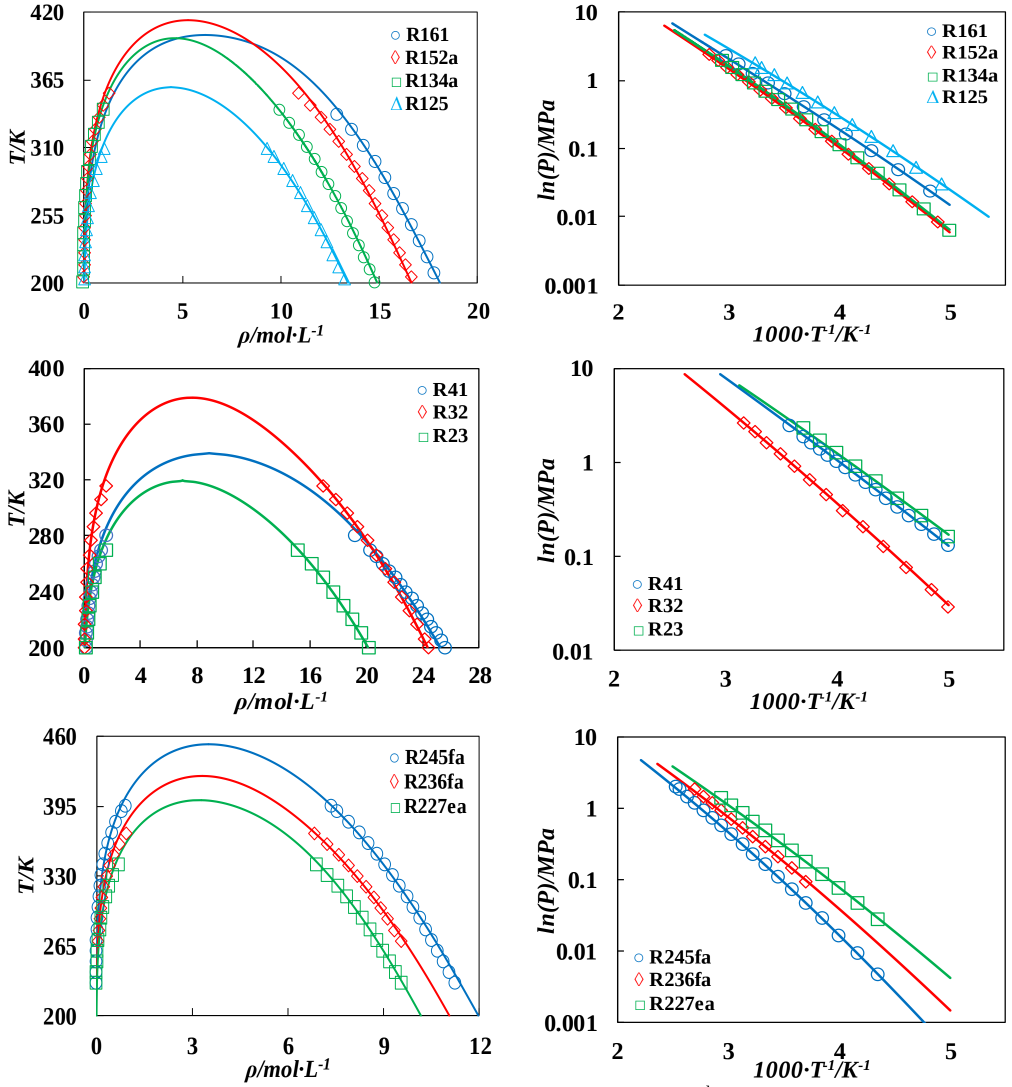


**Fig. S1**. Vapor pressure (right), and coexisting densities (left) for 3rd generation refrigerants studied in this work, with polar soft-SAFT EoS (solid lines), compared to experimental data[2,10] (symbols).

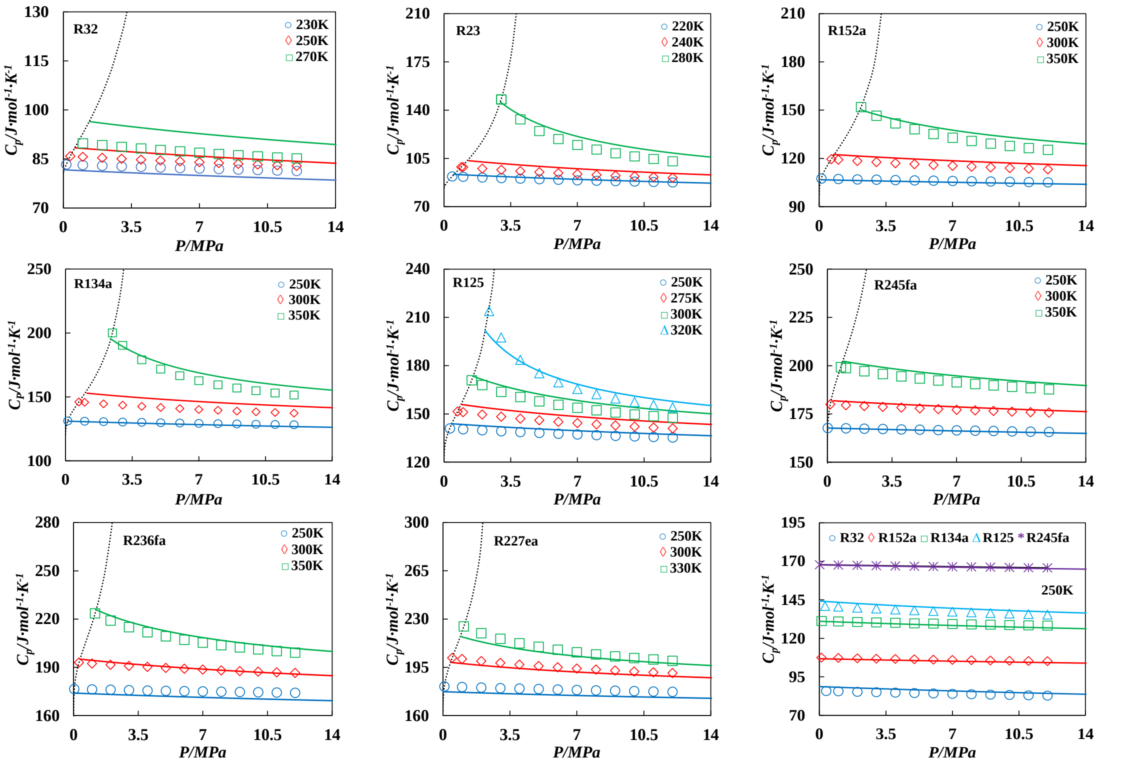


**Fig. S2**. Isobaric heat capacities of 3rd generation refrigerants predicted with polar soft-SAFT EoS (solid lines), compared to experimental data[2,11–13] (symbols).

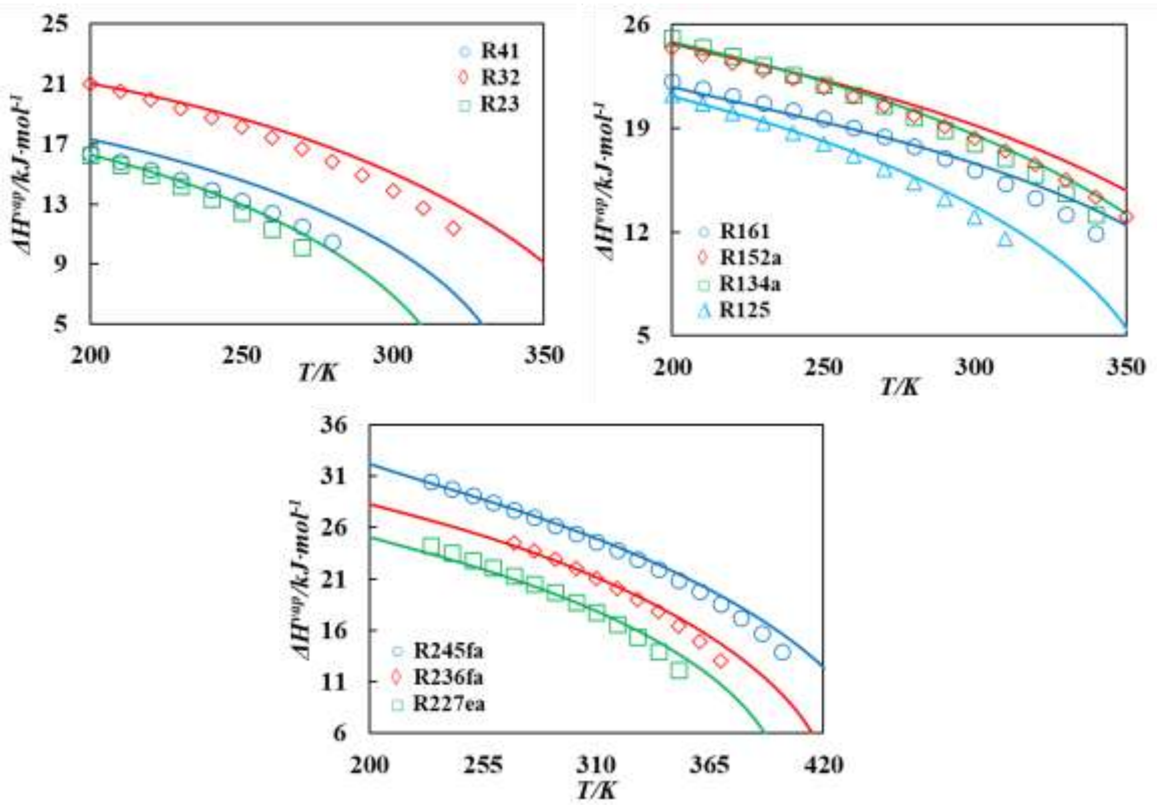


**Fig. S3**. Enthalpy of vaporization of 3rd generation refrigerants predicted with polar soft-SAFT EoS (solid lines), compared to experimental data[2] (symbols).

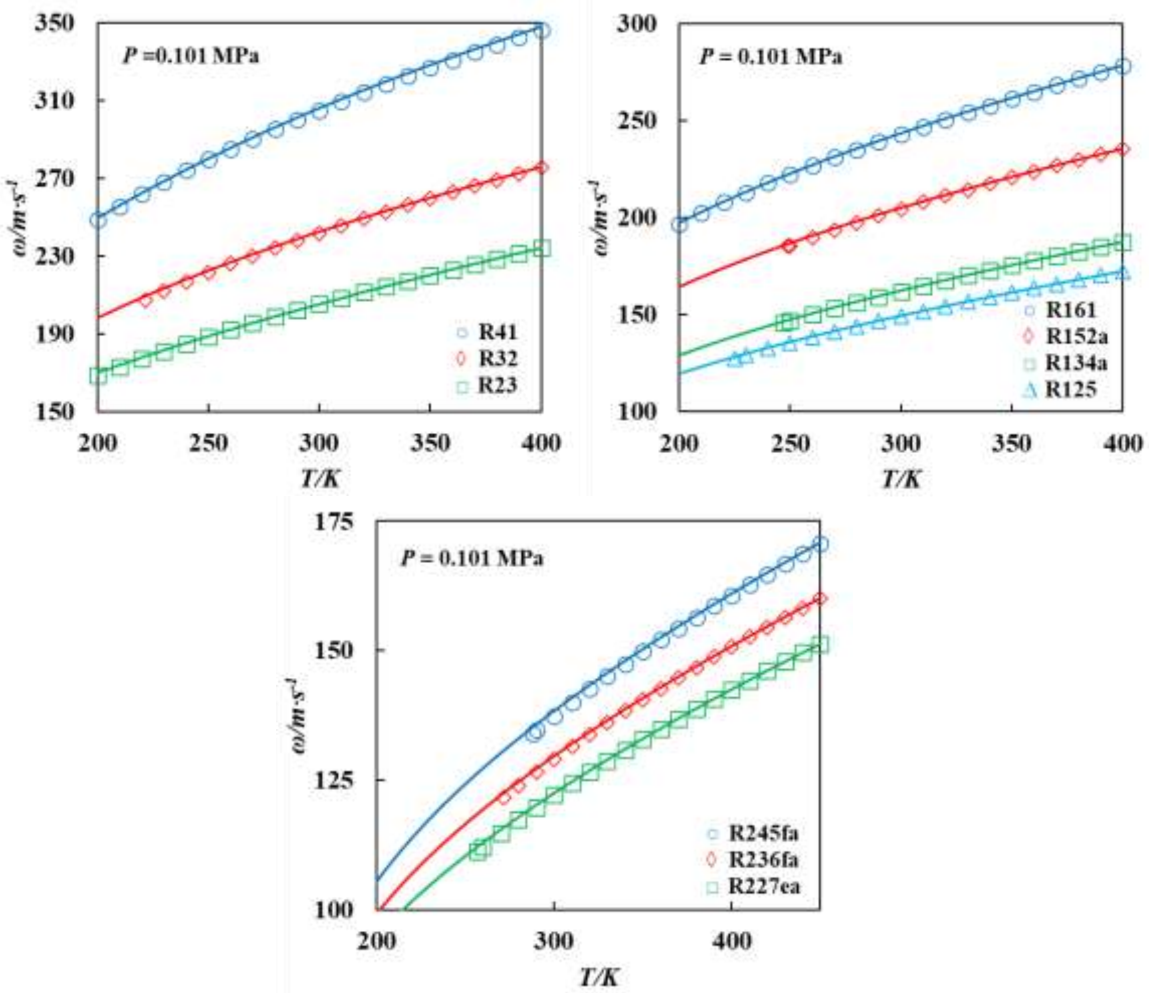


**Fig. S4**. Speed of sound of 3rd generation refrigerants predicted with polar soft-SAFT EoS (solid lines), compared to experimental data[2] (symbols).

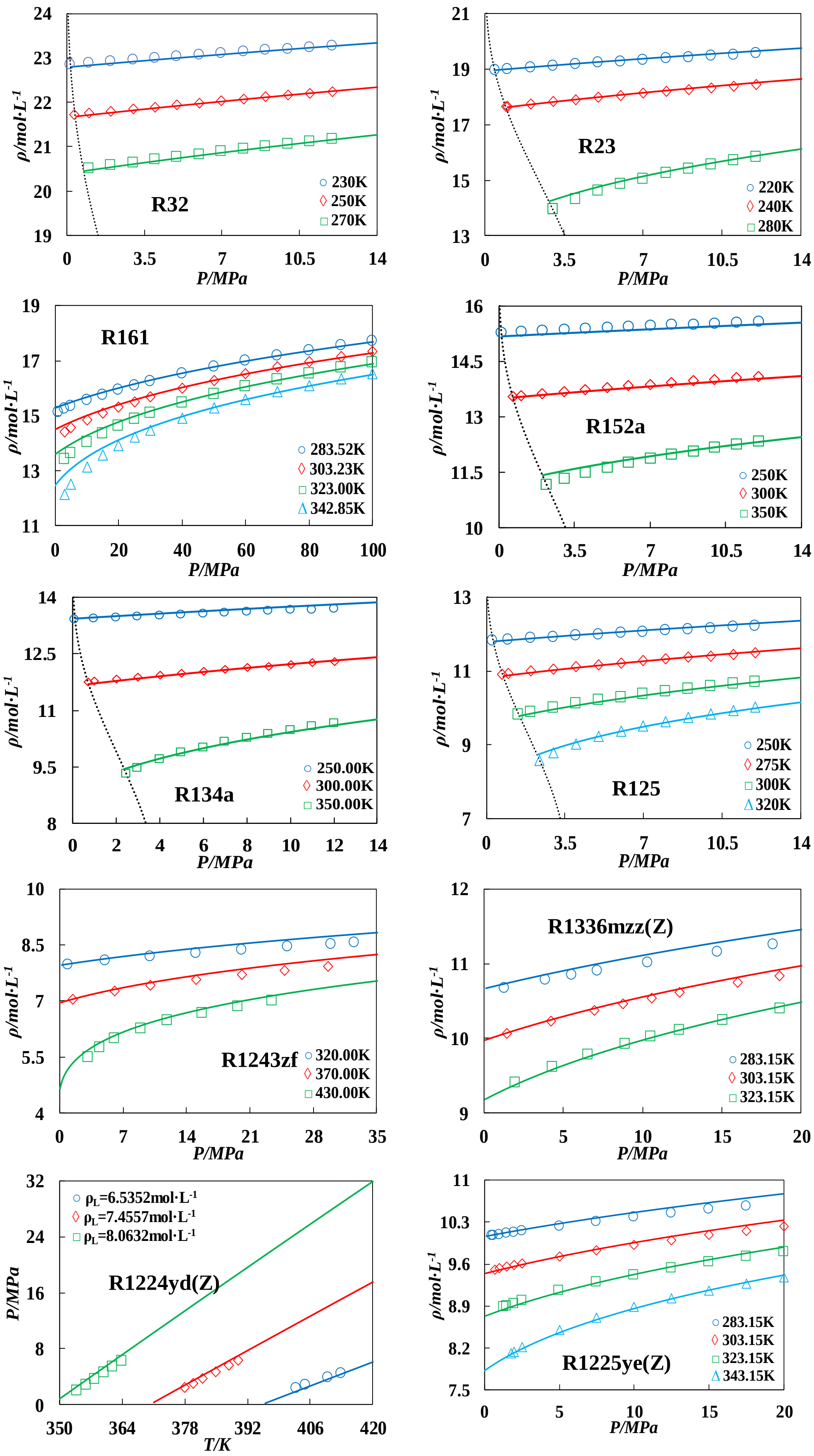


**Fig. S5**. Single-phase density of 3rd and 4th generation refrigerants predicted with polar soft-SAFT EoS (solid lines), compared to experimental data[2,14–18] (symbols).

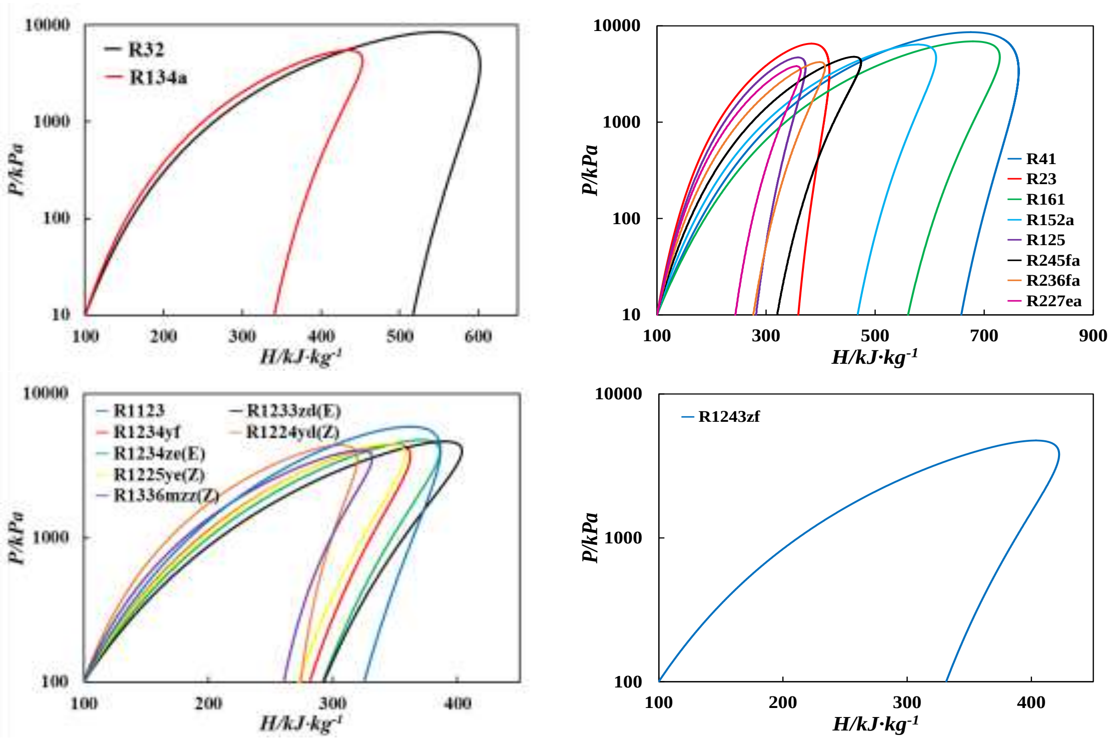


**Fig. S6.** Polar soft-SAFT predicted *PH* diagrams for the eighteen refrigerants modelled in this work.

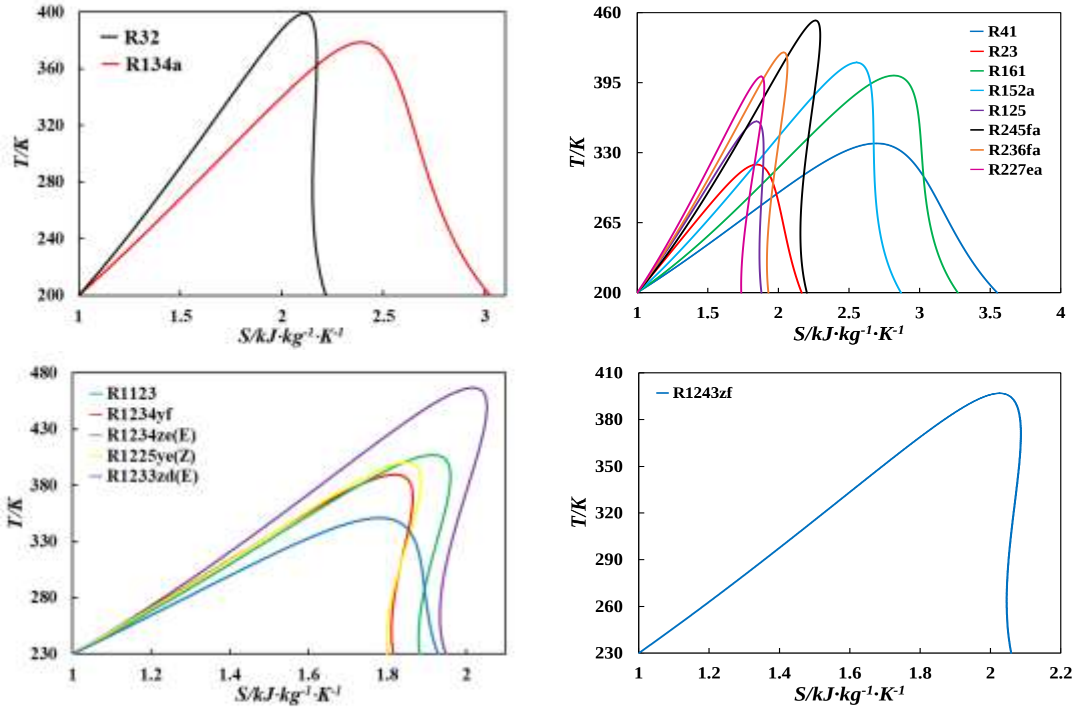


**Fig. S7.** Polar soft-SAFT predicted *TS* diagrams for sixteen refrigerants modeled in this work. Notice, predictions for R1336mzz(Z) and R1224yd(Z) cannot be obtained due to unavailability of ideal gas entropy for these refrigerants.

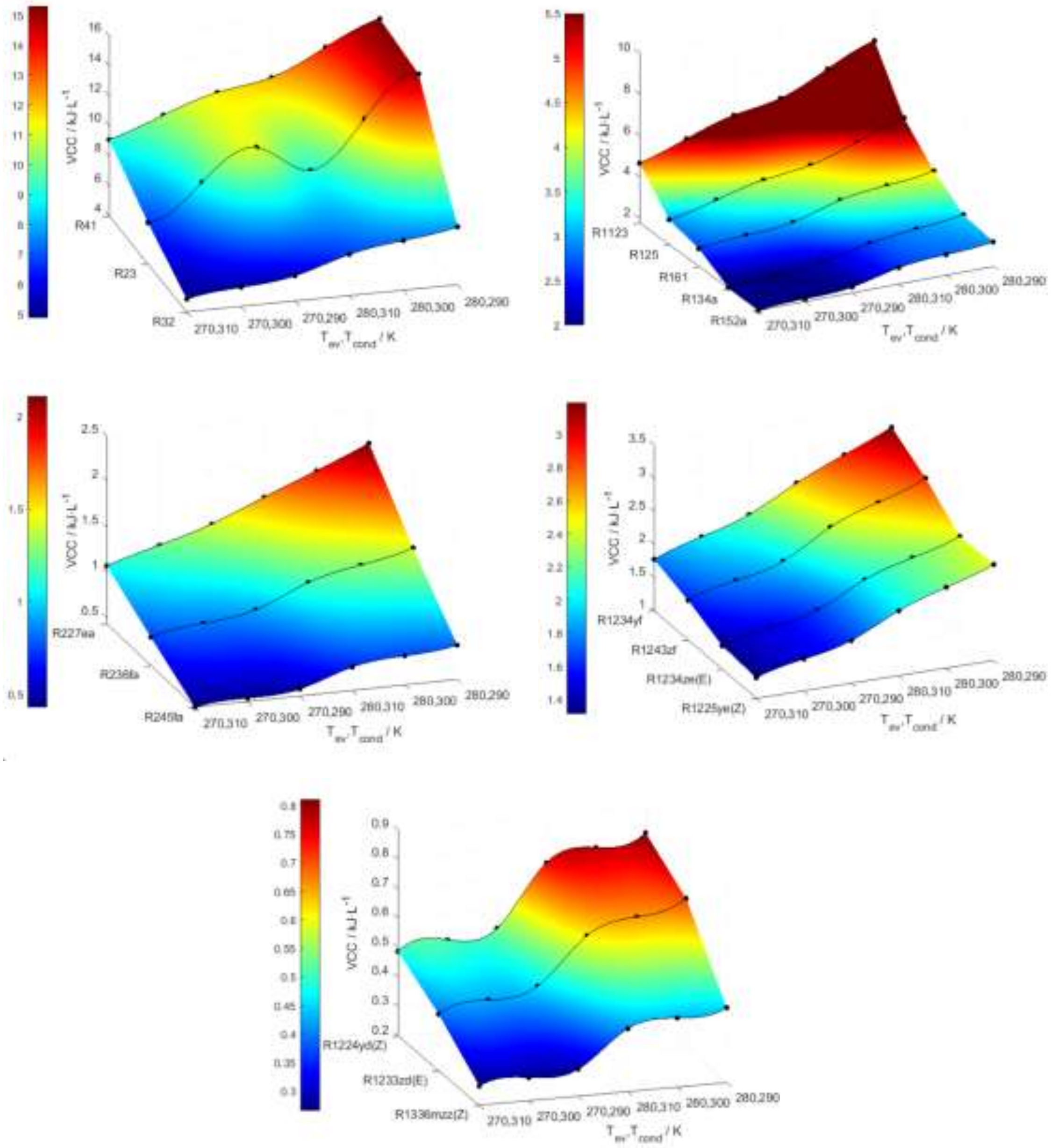


**Fig. S81.** VCC simulated at variable evaporator and condenser conditions for refrigerants modeled in this work

# References


(1) Eyerer, S.; Dawo, F.; Kaindl, J.; Wieland, C.; Spliethoff, H. Experimental Investigation of Modern ORC Working Fluids R1224yd(Z) and R1233zd(E) as Replacements for R245fa. *Appl. Energy* **2019**, *240* (December 2018), 946–963. https://doi.org/10.1016/j.apenergy.2019.02.086.

(2) Lemmon, E. W.; Huber, M. L.; McLinden, M. O. *NIST Reference Fluid Thermodynamic and Transport Properties — REFPROP, Version 9.0, National Institute of Standards and Technology, Gaithersburg, MD*; 2013.

(3) HoneyWell's Genetron Properties software. *HoneyWell's Genetron Properties Software (Version 1.4)*; 2010.

(4) Pinault, J.-L. Anthropogenic and Natural Radiative Forcing: Positive Feedbacks. *J. Mar. Sci. Eng.* **2018**, *6* (4), 146. https://doi.org/10.3390/jmse6040146.

(5) Bobbo, S.; Nicola, G. Di; Zilio, C.; Brown, J. S.; Fedele, L. Low GWP Halocarbon Refrigerants: A Review of Thermophysical Properties. *Int. J. Refrig.* **2018**, *90*, 181–201. https://doi.org/10.1016/j.ijrefrig.2018.03.027.

(6) Yang, J.; Gao, L.; Ye, Z.; Hwang, Y.; Chen, J. Binary-Objective Optimization of Latest Low-GWP Alternatives to R245fa for Organic Rankine Cycle Application. *Energy* **2021**, *217*, 119336. https://doi.org/10.1016/j.energy.2020.119336.

(7) Yang, Z.; Feng, B.; Ma, H.; Zhang, L.; Duan, C.; Liu, B.; Zhang, Y.; Chen, S.; Yang, Z. Analysis of Lower GWP and Flammable Alternative Refrigerants. *Int. J. Refrig.* **2021**, *126*, 12–22. https://doi.org/10.1016/j.ijrefrig.2021.01.022.

(8) Wu, X.; Dang, C.; Xu, S.; Hihara, E. State of the Art on the Flammability of Hydrofluoroolefin (HFO) Refrigerants. *Int. J. Refrig.* **2019**, *108*, 209–223. https://doi.org/10.1016/j.ijrefrig.2019.08.025.

(9) Pàmies, J. C. Bulk and Interfacial Properties of Chain Fluids: A Molecular Modelling Approach. PhD Thesis, Universitat Rovira i Virgili, 2004.

(10) Bell, I. H.; Wronski, J.; Quoilin, S.; Lemort, V. Pure and Pseudo-Pure Fluid Thermophysical Property Evaluation and the Open-Source Thermophysical Property Library CoolProp. *Ind. Eng. Chem. Res.* **2014**, *53*, 2498–2508. https://doi.org/doi.org/10.1021/ie4033999.

(11) Liu, Y.; Zhao, X.; Lv, S.; He, H. Isobaric Heat Capacity Measurements for R1234yf from 303 to 373 K and Pressures up to 12 MPa. *J. Chem. Eng. Data* **2017**, *62* (3), 1119–1124. https://doi.org/10.1021/acs.jced.6b00959.

(12) Liu, Y.; Zhao, X.; He, H.; Wang, R. Heat Capacity of R1234ze(E) at Temperatures from 313 to 393 K and Pressures up to 10 MPa. *J. Chem. Eng. Data* **2018**, *63* (1), 113–118. https://doi.org/10.1021/acs.jced.7b00713.

(13) Liu, Y.; Zhao, X. Measurement of the Heat Capacity of R1233zd(E). *Int. J. Refrig.* **2018**, *86*, 127–132. https://doi.org/10.1016/j.ijrefrig.2017.11.015.

(14) Qi, H.; Fang, D.; Gao, K.; Meng, X.; Wu, J. Compressed Liquid Densities and Helmholtz Energy Equation of State for Fluoroethane (R161). *Int. J. Thermophys.* **2016**, *37* (6), 55. https://doi.org/10.1007/s10765-016-2061-1.

(15) Mclinden, M. O.; Akasaka, R. Thermodynamic Properties of Cis-1,1,1,4,4,4-Hexafluorobutene [R-1336mzz(Z)]: Vapor Pressure, (p, ρ, T) Behavior, and Speed of Sound Measurements and Equation of State. **2020**. https://doi.org/10.1021/acs.jced.9b01198.

(16) Di Nicola, G.; Steven Brown, J.; Fedele, L.; Securo, M.; Bobbo, S.; Zilio, C. Subcooled Liquid Density Measurements and PvT Measurements in the Vapor Phase for 3,3,3-Trifluoroprop-1-Ene (R1243zf). *Int. J. Refrig.* **2013**, *36* (8), 2209–2215. https://doi.org/10.1016/j.ijrefrig.2013.08.004.

(17) Higashi, Y.; Sakoda, N. Measurements of PvT Properties, Saturated Densities, and Critical Parameters for 3,3,3-Trifluoropropene (HFO1243zf). *J. Chem. Eng. Data* **2018**, *63* (10), 3818–3822. https://doi.org/10.1021/acs.jced.8b00452.

(18) Brown, J. S.; Fedele, L.; Di Nicola, G.; Bobbo, S.; Coccia, G. Compressed Liquid Density and Vapor Phase PvT Measurements of Cis -1,2,3,3,3-Pentafluoroprop-1-Ene (R1225ye(Z)). *J. Chem. Eng. Data* **2015**, *60* (11), 3333–3340. https://doi.org/10.1021/acs.jced.5b00562.
